# Characterization of small abdominal aortic aneurysms' growth status using spatial pattern analysis of aneurismal hemodynamics

**DOI:** 10.1038/s41598-023-40139-z

**Published:** 2023-08-24

**Authors:** Mostafa Rezaeitaleshmahalleh, Zonghan Lyu, Nan Mu, Xiaoming Zhang, Todd E. Rasmussen, Robert D. McBane, Jingfeng Jiang

**Affiliations:** 1 Department of Biomedical Engineering, Michigan Technological University, Houghton, MI USA; 2 Joint Center for Biocomputing and Digital Health, Health Research Institute, and Institute of Computing and Cybernetics, Michigan Technological University, Houghton, MI USA; 3 Department of Radiology, Mayo Clinic, Rochester, MN USA; 4 Division of Vascular and Endovascular Surgery, Mayo Clinic, Rochester, MN USA; 5 Department of Cardiovascular Medicine, Mayo Clinic, Rochester, MN USA

**Keywords:** Biomedical engineering, Computational biology and bioinformatics, Cardiology, Fluid dynamics

## Abstract

Aneurysm hemodynamics is known for its crucial role in the natural history of abdominal aortic aneurysms (AAA). However, there is a lack of well-developed quantitative assessments for disturbed aneurysmal flow. Therefore, we aimed to develop innovative metrics for quantifying disturbed aneurysm hemodynamics and evaluate their effectiveness in predicting the growth status of AAAs, specifically distinguishing between fast-growing and slowly-growing aneurysms. The growth status of aneurysms was classified as fast (≥ 5 mm/year) or slow (< 5 mm/year) based on serial imaging over time. We conducted computational fluid dynamics (CFD) simulations on 70 patients with computed tomography (CT) angiography findings. By converting hemodynamics data (wall shear stress and velocity) located on unstructured meshes into image-like data, we enabled spatial pattern analysis using Radiomics methods, referred to as "Hemodynamics-informatics" (i.e., using informatics techniques to analyze hemodynamic data). Our best model achieved an AUROC of 0.93 and an accuracy of 87.83%, correctly identifying 82.00% of fast-growing and 90.75% of slowly-growing AAAs. Compared with six classification methods, the models incorporating hemodynamics-informatics exhibited an average improvement of 8.40% in AUROC and 7.95% in total accuracy. These preliminary results indicate that hemodynamics-informatics correlates with AAAs' growth status and aids in assessing their progression.

## Introduction

An abdominal aortic aneurysm (AAA) is defined as a pathologic dilatation of the abdominal aorta with a diameter exceeding 30 mm^1^. Generally, elective aneurysm repair is considered when an AAA's diameter reaches 55 mm for men or 50 mm for women to prevent rupture^2^. Although average aneurysm growth rates are slow (approximately 2 mm per year), inter-individual growth rates vary considerably, and many AAAs show nonlinear growth^3,4^. Patients with small AAAs undergo serial imaging surveillance to monitor growth status. Since aneurysm growth rate is considered a risk factor^1,5,6^, much research has been devoted to identifying aneurysms that are rapidly growing. Approaches capable of predicting the growth status of aneurysms would be immensely clinically useful and allow us to optimize resource allocation for serial monitoring. For instance, we place patients with a fast-growing AAA under aggressive imaging surveillance or recommend them for early intervention to prevent incident rupture. Therefore, understanding characteristics promoting AAAs’ progress and utilizing them to accurately predict AAA growth has been recognized as a high-priority field of clinical importance^7,8^.

Current guidelines for aneurysm intervention incorporate assessments of diameter, expansion rate, symptoms, and additional risk factors. Generally, the rupture risk is highly correlated to the aneurysm size (i.e., one of the most commonly used morphological parameters). However, small, growing aneurysms still rupture. Conversely, there may be cases where surgical treatment is not necessary for large or rapidly growing aneurysms^7–10^. Unfortunately, adding more morphological indices of AAAs will not generally improve the risk assessments to a satisfactory level^11–13^.

From a mechanobiology perspective, local (disturbed) hemodynamics contributes to the degeneration of the aorta wall and expansion of an AAA^14^. Biologically, disturbed flow promotes the activation of platelets^15–17^ and upregulates inflammatory genes^18,19^. Also, aneurismal vortical flow dictates the bio-transport of activated platelets and inflammatory cytokines (trapped within swirling eddies) to the vascular wall, eventually degrading the vessel wall's structural integrity^20,21^. As a result, studies^22–24^ have been motivated to find factors significantly correlated with AAA growth, including biomechanical factors.

Furthermore, leveraging readily available mechanical learning methods, more than one dozen peer-reviewed studies^12,25–35^ investigated methods for predicting AAAs’ growth; those studies can be divided into two categories. In the first category, seven quantitative studies^12,25–29^ (combining geometric characteristics, biomechanical stresses, and PHI) were able to differentiate AAAs' growth status (i.e., fast- and slowly-growing) in human subjects, and their AUROC values reached between 0.79 and 0.86. In the last category, work^30–35^ has been devoted to quantitatively forecasting AAAs' growth (growth rate and growth curve).

To this end, the objectives of this study are twofold. First, we intend to establish an innovative approach to quantify aneurismal flow disturbance. Although hemodynamics has been long recognized for its importance in vascular remodeling^14^, quantifying the aneurismal flow disturbance has not been advanced. Notably, such a quantification should accurately reflect the degree of the flow disturbance, which plays a vital role in vascular remodeling, as demonstrated in our recent in vitro study^36^. In two recent studies^13,29^, hemodynamic metrics were used to predict AAAs’ growth. Both studies included only a list of summary hemodynamic metrics, e.g., mean WSS, maximum WSS, number of (flow) vortex cores, etc. Because the aneurysmal flow disturbance is spatially complex and varying, quantification of aneurysmal flow disturbance must count for variations in spatial patterns of hemodynamic metrics. Consequently, we advocate a spatial pattern analysis of the hemodynamic parameters to quantify aneurysmal flow disturbance. Second, we explore the usefulness of adopting those spatial pattern analyses of hemodynamics data (i.e., WSS and velocity) for predicting AAAs' growth status (i.e., fast-growing versus slowly-growing) in a "patient-specific" manner.

We proposed two technological innovations enabling the above-mentioned spatial pattern analyses of hemodynamic data. First, we strategically converted WSS data from irregular (computer) meshes between the lumen and blood to image-like data. Second, we developed a novel method representing vector directions of three-dimensional velocity and WSS vectors. Our second innovation involved utilizing an equally partitioned unit sphere, dividing it into 360 partitions, each representing a distinct direction; the unit sphere was partitioned using Leopardi's algorithm^37^. More specifically, by comparing the direction of each WSS and velocity vector with the direction vectors associated with the center point of all 360 partitions, we assigned the WSS vector and velocity vector to the partition exhibiting the closest alignment.

Following the extraction of spatial patterns of WSS and velocity, machine-learning-based predictive modeling was used to demonstrate the usefulness of those spatial patterns extracted from hemodynamics data. Our study is the first report in which automated informatics-like features (hereafter referred to as hemodynamics-informatics) extracted from hemodynamic variables are used for predicting AAA growth status.

## Materials and methods

### Patient cohorts

This study used computed tomography angiography (CTA) images of 80 patients from our internal Mayo Clinic database. All methods in this study were performed in accordance with relevant guidelines and regulations. The institutional review boards at Michigan Technological University (Houghton, MI, USA) and Mayo Clinic (Rochester, MN, USA) approved the study. It was a secondary analysis of existing data, and thus, patient consent was not required by the institutional review board at Mayo Clinic (Rochester, MN, USA).

All patients underwent two CTA scans within a 1-year interval to determine AAA growth rates. The inclusion criteria included: (1) AAA maximum diameters of less than 5.5 cm; (2) patients with sufficient image quality to identify abdominal aorta and iliac arteries; and (3) the availability of contrast-enhanced CTA to delineate both aorta lumen and ILT. Aneurysm size was measured from axial cuts perpendicular to the centerline at the maximal diameter from the anterior to the posterior outer wall.

**Figure 1:**
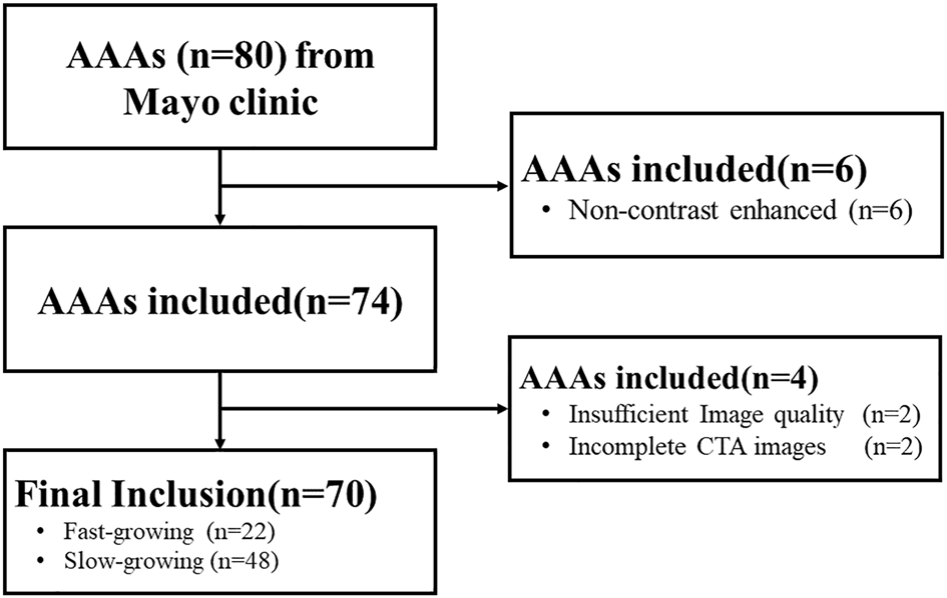
A flowchart showing the data selection. Data acquisition was conducted at Mayo Clinic (Rochester, MN) following a protocol approved by the IRB of Mayo Clinic.

CTA images corresponding to the first CTA scan were utilized to create geometrical models for subsequent computational fluid dynamics (CFD) simulations and morphological analysis. Our data selection process is illustrated in Fig. 1. Aneurysms were then categorized as either fast (≥ 5 mm/year) or slowly growing (< 5 mm/yr), depending on growth rates.

### Morphological and hemodynamic analysis

**Figure 2:**
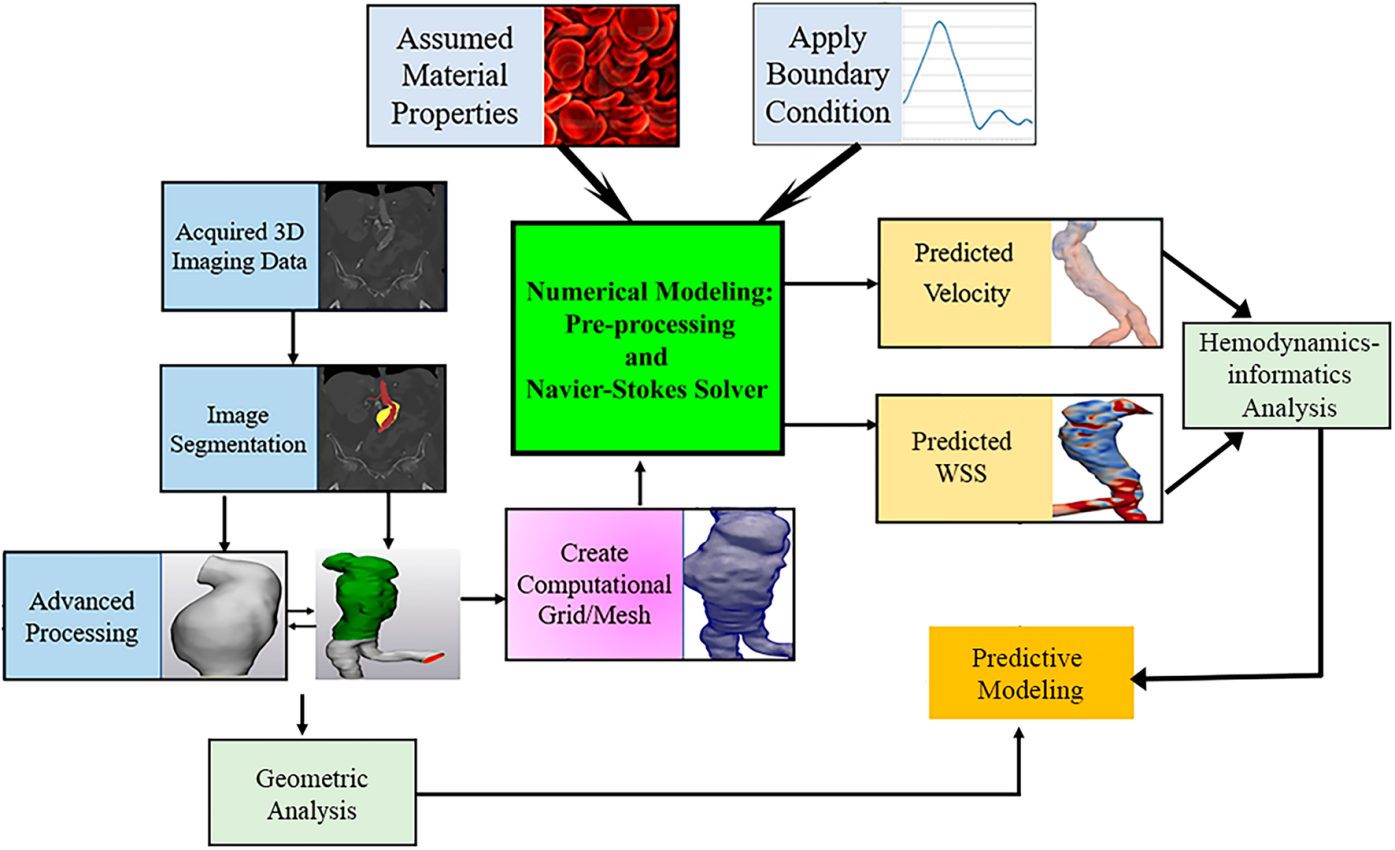
Workflow for acquiring hemodynamic and morphological parameters and performing machine learning-based assessments of AAA growth.

Morphological analysis and CFD simulations are shown in Fig. 2. Each AAA lesion was manually segmented into two regions, lumen and intraluminal thrombosis (ILT), using commercially-available image segmentation software (Mimics Innovation Suite, Version 20, Materialise Inc., Leuven, Belgium). Both lumen and ILT surfaces were smoothed, and some small but disconnected vessels were eliminated using the 3-Matic software (version 18, Materialise Inc., Leuven, Belgium) before geometrical analysis and CFD simulations.

The extracted lumen geometry was meshed and subsequently utilized for computational fluid dynamics (CFD) simulations. Upon obtaining clean vessel geometries, cylindrical flow extensions with a minimum size of 6 times the vessel diameter were added to all inlets and outlets using the open-source Vascular Modelling Toolkit (VMTK) package (version 1.4, www.vmtk.org). To create unstructured 3D tetrahedral meshes with five boundary layers, we employed TetGen (Version 1.4.2)^38^, an open-source mesh generator. Our models typically consisted of a range of 6 to 10 million elements, with an average of approximately 7 million elements. The average element size is roughly 0.03 mm^3^. Mesh sensitivity analysis was conducted to ensure appropriate mesh density was used for this study. A summary of mesh sensitivity is provided in the Supplementary Material.

Subsequently, a commercial CFD solver (Version 21, Fluent, Ansys Inc., PA, USA) was used to solve transient Navier–Stokes equations to compute blood flow velocity and WSS. Blood was modeled to be an incompressible, laminar, Newtonian fluid with a dynamic viscosity of 0.004 Pa·s and a mass density of 1040 $kg/{m}^{3}$. Vessel walls were assumed rigid with a no-slip boundary condition. Following a published report^39^, a pulsatile flow (rate) waveform within the abdominal aorta was implemented as the inlet boundary condition. At the outlets, zero-pressure boundary conditions were prescribed during the CFD simulations. Four cardiac cycles were simulated at 1000 steps per period (0.001 s/timestep) with 20 constant interval data points. More details of quantitative analysis of AAAs' morphology and hemodynamics data can be found in Supplementary Materials and are similar to our recent publication^12^. The CFD workflow was verified with both phase-contrast magnetic resonance angiography (PC-MRA)^40,41^ and ultrasound Doppler^42^ for aneurismal flow.

### Spatial patterns in hemodynamics data

Image features were extracted from medical imaging data using Radiomics software^10^. This process is also known as textural analysis, and conceptually, image features extracted through textural analysis represent spatial patterns of varying intensity values on medical imaging data. Thus, spatial pattern analysis of hemodynamics data was conducted using an open-source software package (PyRadomics^43^). Recall that both velocity and WSS data were located on unstructured mesh, and spatial pattern analysis by PyRadiomics can be performed only for uniformly sampled data (i.e., image or image-like data). Converting the original hemodynamics data on unstructured meshes into image-like hemodynamics data enabled the proposed spatial pattern analyses, as described below. Hereafter, we refer to our technologies as "hemodynamics-informatics" to distinguish our technology from conventional Radiomics-type studies for imaging data. More specifically, hemodynamics-informatics includes velocity-informatics (i.e., spatial pattern analyses of velocity data) and WSS-informatics (i.e., spatial pattern analyses of WSS data).

Hemodynamics-informatics was calculated using first-order, second-order, and higher-order statistics^43,44^ available in PyRadiomics. First-order features represent the voxel intensity distribution using mean, median, and maximum. Second-order statistic metrics are computed based on an inter-relationship between adjacent voxel intensity: Gray Level Co-occurrence Matrix (GLCM)^45^, Gray Level Run Length Matrix (GLRLM)^46^, and Gray Level Size Zone Matrix (GLSZM). Filtering or mathematical transform (e.g., Laplacian of Gaussian filter or wavelet transform) is first applied to the original imaging data to calculate higher-order statistics features. Such a filter or transform reduces noise, and the resultant low-noise image data is used to identify repetitious (spatial) patterns using first-order or second-order statistics. For completeness, a brief introduction to second-order and higher-order statistics is provided in the Appendix.

### Velocity-informatics

**Figure 3:**
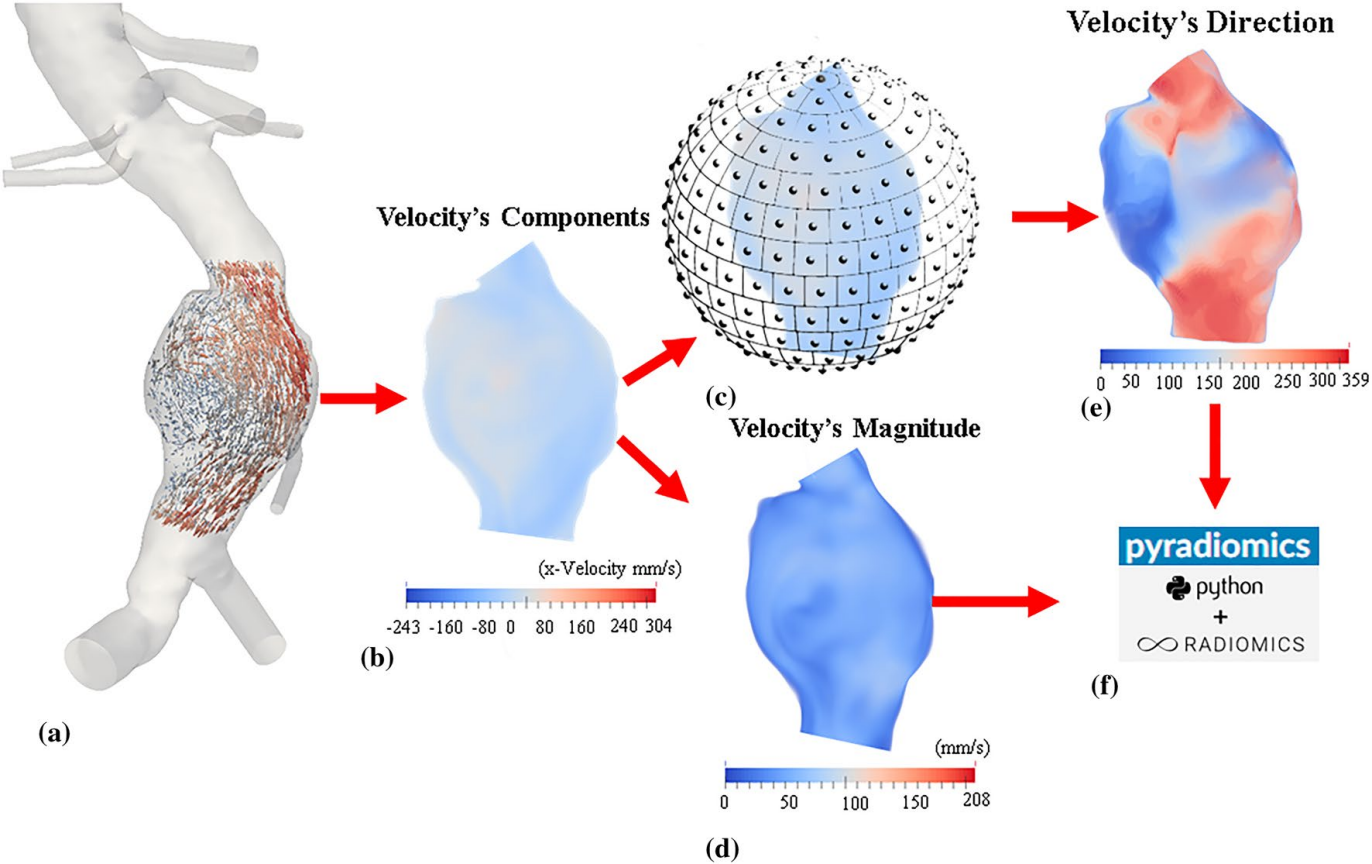
A graphic illustration of procedures involved in calculating velocity-informatics: (**a**) Isolate an aneurysmal velocity field residing on an unstructured grid (**b**) Resample all components of velocity to a uniform grid (**c**) Map velocity to a partitioned sphere (**d**) Generate three-dimensional (3D) velocity magnitude (MVelocity) image (**e**) Obtain 3D velocity Direction (DVelocity) image (**f**) Using MVelocity and DVelocity as 3D images in PyRadiomics for assessment.

This study used a 3D velocity field only at the peak systole (Fig. 3a). All velocity vectors at the peak systole within the AAA were first isolated (Fig. 3b). Then, all identified velocity vector components were resampled (using interpolation) to a uniform grid. Both magnitude and direction velocity data were converted to image-like data. More specifically, 5–95% magnitude velocity data were mapped to normalized magnitude velocity data (Fig. 3d). For each velocity vector, its directional vector can be mapped to one of the regions on an equally partitioned unit sphere^37^ (360 regions in this study; Fig. 3c) as follows. First, each region of the unit sphere (see Fig. 3c) represents a directional vector (hereafter referred to as a regional vector).

The regional vector is calculated by connecting the center of the unit sphere to the centroid of the region. Then, we assign each directional velocity vector to a region whose regional vector is most closely aligned with the directional velocity vectors (i.e., the smallest angular difference between the regional vector and directional velocity vector). Once this process is completed, we have two 3D image-like velocity data: Velocity's Direction (Fig. 3d) and Velocity's Magnitude (Fig. 3e) images. Finally, both magnitude and velocity images are fed into an open-source Python package (PyRadiomics, version 1.4) to extract spatial patterns (Fig. 3f).

### WSS-informatics

**Figure 4:**
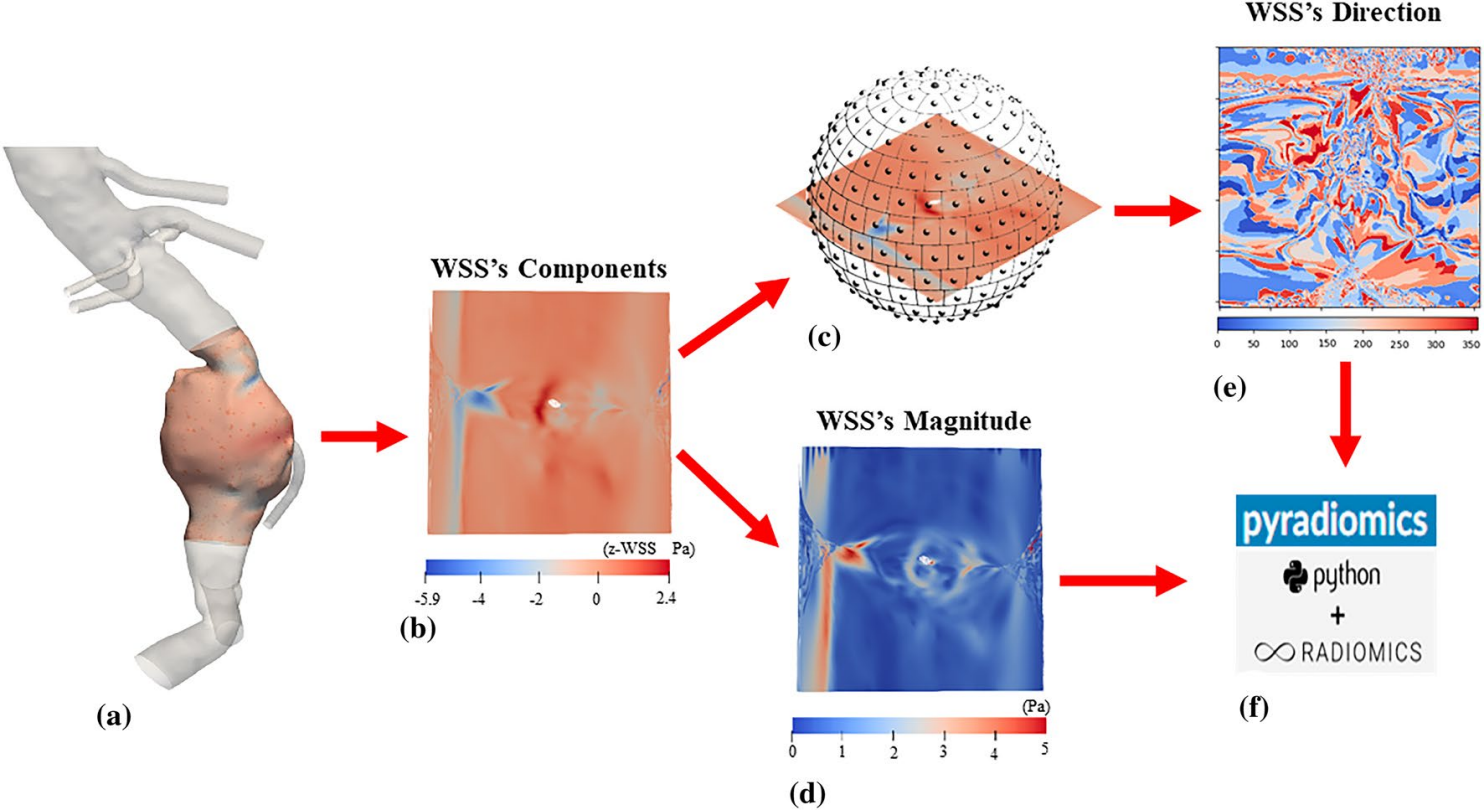
A graphic illustration of procedures involved in calculating WSS-informatics: (**a**) Isolate an aneurysmal WSS field (**b**) Unwrap 3D WSS geometry to 2D map and then Resample all components of WSS to a uniform grid (**c**) Map WSS to an equally-partitioned unit sphere (**d**) Obtain WSS magnitude (MWSS) image (**e**) Obtain WSS Direction (DWSS) image (**f**) Using MWSS and DWSS as 2D images in PyRadiomics.

WSS-informatics features were also calculated from the WSS data at the peak systole. Our established workflow for WSS-informatics is shown in Fig. 4.

As shown in Fig. 4a, we first identified WSS data at the peak systole within the extent of the AAA. Because WSS data was located at the interface between the lumen and blood, which was made by triangles with varying sizes, a conformal mapping technique^47,48^ was used to convert the original WSS data to image-like data in 2D (see the 2D planar surface in Fig. 4b). Following a similar procedure described for Velocity-informatics, magnitude and directional WSS data on the 2D planar surface can be calculated. Corresponding results are displayed in an image format (see Fig. 4d and e). Both image-like WSS data were fed into PyRadiomics to extract spatial patterns/features related to the magnitude and directional WSS data.

### Dimensionality reduction and feature selection

Velocity-informatics, WSS-informatics, morphological, and conventional hemodynamic analyses were performed in this study, resulting in a large number of available parameters (A total of 845 different parameters). To avoid overfitting in the subsequent machine-learning-based predictive modeling, feature selection and dimensionality reduction were conducted using R software (R-studio, version 1.3). Specifically, a stepwise process was implemented to select the features as follows. First, all features were assessed by the Wilcoxon Rank Sum Test^49^ to determine features that significantly differentiate between slowly- and fast-growing aneurysms. Second, features with a p-value > 0.8 were removed from further consideration. Third, VarImp (variable importance function) from the Caret package in the R software was utilized for feature selection by performing a tenfold cross-validation. Feature selection has been repeated over 100 realizations and ranked from 1 to the number of selected features in a particular realization.

**Figure 5:**
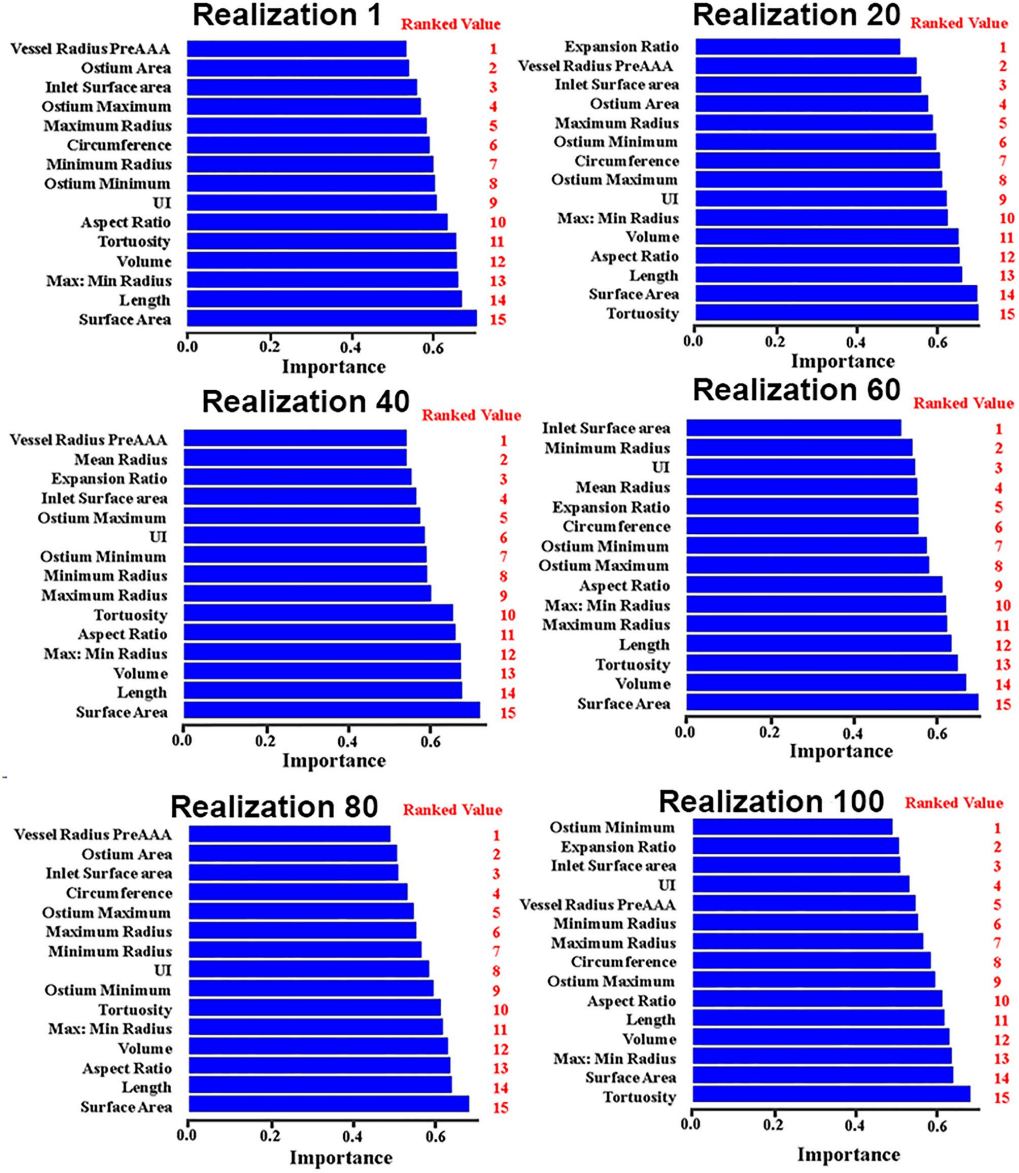
Bar plots illustrating changes in selected features during 100 realizations. The x-axis represents the importance of the variables, and the y-axis represents the names of the features. Ranked features were based on VarImp (variable importance function) from the Caret package in the R software.

One example of this process is shown in Fig. 5. During the data processing, the list of top-15 features was reasonably stable during four randomly selected realizations.

### Predictive modeling of AAAs' growth status

Selected features were taken into support vector machine^50^ (SVM, R Studio, version 1.3) to construct the optimized models for predicting AAAs' growth status. During the SVM training, 90% of the data were randomly selected as training data, while the remaining 10% of the data were used for testing. During training, tenfold cross-validation was used to finalize a selected SVM model that will be applied to testing data to evaluate the performance of the selected SVM model. This process was repeated 100 times to ensure that the results were statistically stable.

To demonstrate the merits of hemodynamics-informatics, the performance of SVM was compared with five other commonly used machine-learning predictive methods: Lasso, K-nearest neighbor (KNN), random forest (RF), and Gradient Boosting Method (GBM). Comparing prediction performance among six machine-learning methods demonstrates that the elevated performance due to the inclusion of hemodynamics-informatics metrics is sustainable (i.e., not specific to a particular machine-learning algorithm).

### Ethics approval and consent to participate

This study was approved by Institutional Review Boards at Michigan Technological University and Mayo Clinic. Because this is a secondary analysis of existing imaging data, patient consent was not required.

## Results

### Demographics and clinical information

A total of 70 AAA cases with an initial Maximum Diameter ranging from 28 to 70 mm were used in this study. 22 (31.42%) of cases belonged to patients with fast-growing aneurysms. The demographics and clinical information of the study population are summarized in Table 1.

**Table 1:** Comparison of clinical features between fast- and slowly-growing AAAs.

Variable	Total n = 70		P-value
	Slow-growing	Fast-growing	
No.of patient	48 (68.6%)	22 (31.40%)	NA
Age in year, mean($\pm$ SD)	80.75 $\pm 9.64$	79.57 $\pm$ 8.65	0.63
Male gender, N (%)	39/48 (81.25%)	18/22 (81.82)	0.96
BMI($\mathrm{kg}/{\mathrm{m}}^{3}$)	28.81 $\pm$ 5.72	29.19 $\pm 4.87$	0.97
Cardiovascular risk factor			
History of hypertension	44/48 (91.67%)	20/22 (90.91%)	0.92
History of smoking	41/48 (83.67%)	16/22 (76.19%)	0.55
Diabetes	11/48 (22.92%)	05/22 (22.73%)	0.99
Dyslipidemia	21/48 (81.25%)	21/22 (95.45%)	0.12
Coronary artery disease	25/48 (52.08%)	13/22 (59.09%)	0.59
Carotid artery disease	38/48 (79.17%)	18/22 (81.82%)	0.80
Family history of aneurysm	04/48 (8.51%)	03/22 (13.64%)	0.50
Medication			
Antiplatelet (Aspirin)	40/48 (83.33%)	17/22 (77.27%)	0.55
Antihypertension (Diuretics, Beta blocker, Ace Inhibitors, Calcium channel blocker, Angiotensin receptor blockers)	40/48(83.33%)	21/22 (95.45%)	0.16
Statins	38/48 (79.17%)	16/22 (72.73%)	0.86
AAA volume	87.73 $\pm$ 26.55	75.18 $\pm 39.36$	0.15

Unlike recent studies^51^, there were no significant differences between the fast- and slowly-growing AAAs in age, sex, cardiovascular risk factor, history of coronary artery and carotid artery disease, family history of aneurysm, and medication usage (all p > 0.05). Based on our feature selection method explained earlier, medication usage of Beta blockers, Diuretics, and Calcium channel blockers and patients with coronary artery disease and Dyslipidemia were recognized as relevant clinical factors in the prediction of AAAs’ growth status. Lumenal volume for fast-growing AAAs was, on average, 16% higher than for slowly-growing AAAs, and the presence of ILT was common in both slowly- and fast-growing AAAs.

### Dimensionality reduction and features selection

Following the protocol described for Dimensionality Reduction and Feature Selection, the 18 most important features of Geometry-lumen, Geometry-lumen + ILT, velocity-informatics (magnitude and direction), and WSS-informatics (magnitude and direction) are listed in Tables 2, 3, and 4.

**Table 2:** A list of AAAs’ geometric parameters was selected as essential features and tested in predictive modeling.

Category	Variables	Slowly-growing	Fast-growing	P-value
Geometry_lumen	*Surface area (mm^2^)	9936.74 $\pm$ 3517.85	11,423.12 $\pm 2369.46$	0.05
	Tortosity	1.22 $\pm 0.08$	1.20 $\pm 0.11$	0.45
	Volume (mm^3^)	87,188.$21\pm 27780.89$	75,186.82 $\pm 39654.71$	0.15
	Length(mm)	110.07 $\pm 21.96$	101.11 $\pm 24.10$	0.09
	Max:Min Radius	18.23 $\pm 3.21$	17.41 $\pm 4.04$	0.19
	Aspect ratio	5.16 $\pm 1.45$	6.11 $\pm 6.52$	0.51
	UI	0.79 $\pm 0.11$	0.82 $\pm 0.10$	0.20
	Maximum Radius (mm)	18.23 $\pm 3.21$	17.41 $\pm 4.04$	0.39
	Ostium minimum (mm)	8.70 $\pm 2.03$	9.67 $\pm 2.03$	0.08
Geomtry_(lumen + ILT)	Surface Area_t (mm^2^)	15,247.15 $\pm 4171.63$	12,813.94 $\pm 4252.48$	**0.01**
	Volume_t (mm^3^)	151,181.36 $\pm 65708.45$	118,755.11 $\pm 56963.86$	**0.02**
	Length_t (mm)	115.88 $\pm 24.12$	106.48 $\pm 24.42$	0.09
	Maximum radius_t (mm)	23.95 $\pm 3.53$	22.21 $\pm 4.03$	0.06
	NTT2	0.36 $\pm 0.10$	0.32 $\pm 0.10$	0.13
	Aspect ratio_t	4.62 $\pm 1.38$	3.96 $\pm 0.86$	**0.02**
	Volume_t-Volume (mm^3^)	69,236.91 $\pm 70582.50$	43.568.28 $\pm 39397.53$	**0.04**
	NTTD	0.80 $\pm 0.19$	0.73 $\pm 0.19$	0.16
	Max:Min radius_t	2.41 $\pm 0.88$	2.56 $\pm 0.66$	0.44

Geometric parameters are defined in Supplementary Materials.

Parameters listed as Mean ± one standard deviation and variables with p-value < 0.05 are in bold.

*NTT2* Normalized thrombosis thickness 2, *NTTD* Normalized thrombosis thickness Differences.

NTT2 and NTTD are described in Supplementary Materials. UI denotes the undulation index. A subscript t indicates that the variable is calculated from an AAA geometry with ILT.

**Table 3:** A list of AAAs’ WSS-Informatic parameters was selected as essential features and tested in predictive modeling.

Category	Variables	Slowly-growing	Fast-growing	P-value
MWSS-informatics	Autocorrelation	28.60 $\pm 26.54$	13.69 $\pm 11.97$	**0.02**
	Joint average	4.97 $\pm 2.47$	3.21 $\pm 1.66$	**0.02**
	Sum average	9.39 $\pm 4.94$	6.43 $\pm 3.31$	**0.02**
	High gray level emphasis	5.95E4 $\pm 9.4$ E5	2.42E5 $\pm$ 4.1E5	0.19
	Low gray level run emphasis	0.14 $\pm 0.18$	0.20 $\pm 0.22$	**0.03**
	Large area low gray level emphasis	5.41E7 $\pm 3.09$ E7	4.06E7 $\pm$ 1.13E7	0.08
	Cluster shade	10.54 $\pm 21.07$	18.83 $\pm 28.73$	0.06
	Long run low gray level	2101.69 $\pm 6356.45$	3468.55 $\pm 8824.43$	0.09
	High gray level zone emphasis	36.46 $\pm 18.15$	28.21 $\pm 7.74$	0.12
DWSS-informatics	Large area low gray level emphasis	554.76 $\pm 543.85$	340.39 $\pm 195.86$	0.11
	Sum entropy	4.28 $\pm 0.24$	4.37 $\pm 0.15$	0.13
	Maximum probability	0.11 $\pm 0.03$	0.10 $\pm 0.02$	0.13
	Long run low gray level emphasis	2.18 $\pm 1.14$	1.73 $\pm 0.49$	0.12
	Joint entropy	5.41 $\pm 0.41$	5.51 $\pm 0.32$	0.31
	Gray level nonuniformity	1756.96 $\pm 262.67$	1689.15 $\pm 248.58$	0.27
	IMC2	0.99 $\pm 0.01$	0.98 $\pm$ 0.01	0.15
	Run variance	16.89 $\pm 8.38$	14.26 $\pm 5.23$	0.22
	Zone entropy	7.47 $\pm 0.56$	7.63 $\pm 0.55$	0.25

Parameters listed as Mean ± one standard deviation and variables with p-value < 0.05 are in bold.

WSS-informatics parameters are described in Appendix.

**Table 4:** A list of AAAs’ Velocity-Informatic parameters was selected as essential features and tested in predictive modeling.

Category	Variables	Slowly-growing	Fast-growing	P-value
Mvelocity-informatics	wavelet.HLH.median	2.34E − 02 $\pm 0.16$	7.61E − 04 $\pm$ 0.01	0.51
	SumSquares	3.28 $\pm 1.39$	2.46 $\pm 1.58$	**0.03**
	wavelet.HLH.Kurtosis	192.80 $\pm 227.81$	349.96 $\pm 348.70$	**0.03**
	wavelet.HLL.Entropy	1.02 $\pm 0.19$	0.99 $\pm$ 0.01	0.47
	wavelet.LHH.Median	− 5.50E − 04 $\pm 0.00$	1.54E − 03 $\pm 0.00$	** < 0.01**
	ClusterTendency	12.87 $\pm 5.62$	9.73 $\pm 6.27$	**0.04**
	Autocorrelation	18.66 $\pm$ 11.84	12.97 $\pm 8.79$	0.05
	HighGrayLevelRunEmphasis	15.47 $\pm 8.24$	11.48 $\pm 5.88$	0.05
	GrayLevelVariance	3.56 $\pm 1.37$	2.77 $\pm 1.53$	**0.03**
Dvelocity-informatics	Wavelet.LLH.Minimum	− 6.98E − 03 $\pm 0.02$	− 1.88E − 02 $\pm 0.03$	0.11
	MaximumProbability	0.35 $\pm 0.12$	0.25 $\pm 0.10$	** < 0.01**
	JointEnergy	13.32 $\pm$ 1.38	12.65 $\pm 1.32$	0.06
	Wavelet.HHH.Skewness	− 0.24 $\pm$ 0.35	0.00 $\pm 0.21$	** < 0.01**
	Wavelet.HLL. 90Percentil	4.15 $\pm$ 2.30	5.77 $\pm 2.22$	** < 0.01**
	Wavelet.LLL. Uniformity	0.13 $\pm 0.08$	0.07 $\pm$ 0.04	** < 0.01**
	Log.sigma.1.0.mm.3D.90Percentile	3.05 $\pm$ 1.50	4.23 $\pm 1.45$	** < 0.01**
	GrayLevelNonuniformity	540.48 $\pm$ 758.20	253.04 $\pm$ 131.11	**0.08**
	LongRunHighGrayLevelEmphasis	73,534.3 $\pm 67768$	34,791.3 $\pm$ 265.5	**0.01**
	JointEntropy	3.34 $\pm 0.80$	3.90 $\pm$ 0.63	** < 0.01**

Parameters listed as Mean ± one standard deviation and variables with p-value < 0.05 are in bold.

H and L indicate high-pass and low-pass filters in any direction. Velocity-informatics parameters are described in Appendix.

### Model assessment

The twelve best SVM models (with linear kernels) and their performances can be found in Tables 5 and 6.

**Table 5:** Models created using a combination of parameters in six categories.

Model #	Note on variable used	Metrics
1	Geomtry___lumen	Surface area + Volume + Tortusity + Max:Min Radius + Aspect Ratio + UI Ostium min Radius + Parent Vessel Circumference
2	Flow Vortices Only	Vortex Core + TA_WSS_Minimum
3	WSS only	SA_OSI
4	WSS-informatic only	Kurtosis.DWSS + JointEntropy.DWSS + LargeAreaLowGraylevelEmphasis.DWSS + Sum Entropy.DWSS
5	Velocity-informatic only	GrayLevelNonUniformity.Dvelocity + RunVariance.Dvelocity + SumEntropy.D.velocity + ClusterProminence.Dvelocity + ClusterShade.Dvelocity
6	Geomtry___lumen + PHI	Model 1 + Antihypertensive Medication Usage (Beta blockers + Diuretics) + Coronary artery disease
7	Geomtry___lumen + Geomtry___(lumen + ILT) + PHI	Suface Area_t_ + Model6-{Surface Area + Length + Volume}
8	Geomtry___lumen + Geomtry___(lumen + ILT) + PHI + WSS	**Model 7 + **TA_WSS_Max
9	Geomtry___lumen + Geomtry___(lumen + ILT) + PHI + Flow Vortices	**Model 7 + **TA_DVO
10	Geomtry___lumen + Geomtry___(lumen + ILT) + PHI + WSS-informatic	**Model 7** -{Tortusity + Aspect Ratio} + GrayLevelNonuniformity.DWSS + LargeArealowGraylevelEmphasis.DWSS
11	Geomtry___lumen + Geomtry_(lumen + ILT) + PHI + Velocity-informatic	**Model 7** -{Tortusity + Aspect Ratio} + GrayLevelNonuniformity.DVelocity
12	Geomtry___lumen + Geomtry___(lumen + ILT) + PHI + WSS-informatic + Velocity-informatic	**Model 7** -{Tortusity + Aspect Ratio} + GrayLevelNonuniformity.DVelocity + LargeArealowGraylevelEmphasis.DWSS

Subscript t represents morphological indices related to Geomtry___(lumen + ILT), brackets contain variables subtracted from Model 7.

*Dvelocity* directional velocity, *DWSS* Directional WSS, *SA-OSI* Spatially Averaged Oscillatory Shear Index., *TA-WSSMin* Temporally Averaged Wall Shear Stress Minimum, *TA-DVO* Temporally Averaged Degree of Volume Overlap.

**Table 6:** AUROC and accuracy calculated for all predicted models using AAA geometry, hemodynamics, PHI, WSS-informatics, and velocity-informatics.

Model #	AUROC	Total accuracy	Fast-growing accuracy(%)	Slow-growing accuracy (%)
Model 1	0.74 (0.69–0.78)	67.30	14.50	93.75
Model 2	0.53 (0.50–0.58)	66.67	0.00	**100.00**
Model 3	0.57 (0.46–0.58)	66.67	0.00	**100.00**
Model 4	0.66 (0.63–0.70)	64.83	0.50	97.00
Model 5	0.76 (0.72–0.79)	68.83	50.50	78.00
Model 6	0.82 (0.78–0.85)	78.00	54.00	88.75
Model 7	0.86 (0.83–0.89)	78.50	58.00	88.75
Model 8	0.85 (0.82–0.88)	77.80	55.50	89.00
Model 9	0.85 (0.82–0.88)	76.10	52.50	88.00
Model 10	0.88 (0.87–0.89)	81.00	69.50	86.75
Model 11	**0.93 (0.90–0.95)**	**87.83**	**82.00**	90.75
Model 12	**0.93 (0.90–0.95)**	83.10	72.00	88.75

Data in parenthesis shows the confidence interval of calculated AUC over 100 iterations for any models.

The number in bold indicates the best-estimated results within 12 models.

In the first five models, the parameters related to lumen morphology, WSS, flow vortices, WSS-informatics, and velocity-informatics were assessed for their individual usefulness in predicting the growth status of AAAs. Notably, two models (Models 1 and 5) associated with the lumen morphology and velocity-informatics demonstrated superior performance among all five models. Recall that one of the objectives of this study is to assess the impact of incorporating hemodynamics-informatics on enhancing prediction outcomes. Hence, Model 1 was established as the baseline model for predicting the growth status of AAAs.

In Model 6, PHI information was added to Geometry_lumen. This combination led to a mean area under the receiving operator curve (AUROC) of 0.82 and a total accuracy of 78.00%, with 54.00% and 88.75% fast- and slowly-growing identification. In Model 7, the ILT geometric information was added to Model 6, which improved AUROC and mean total accuracy by 4% and 0.5%, respectively.

**Figure 6:**
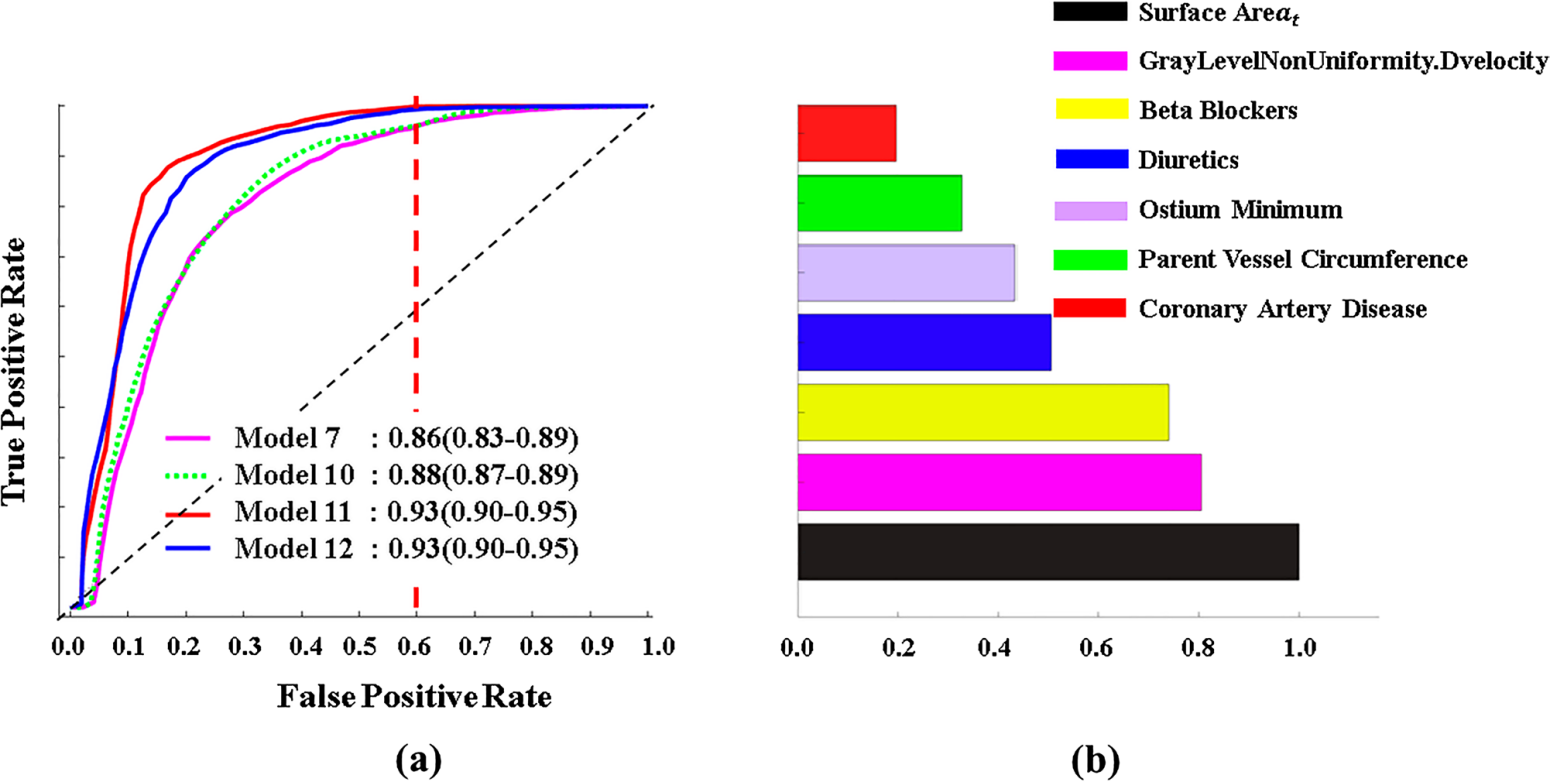
The average ROC curve over 100 iterations in SVM and the most important features in predictions: (**a**) The Pink line is the model containing Geomtry_lumen + Geomtry_(lumen + ILT) and PHI. Dot green is a model made up of Model 7 and WSS-informatics variables. Moreover, the blue line has Model 7 and velocity-informatics variables. The perpendicular dashed red line specifies the cutoff by which Models 11 and 12 can correctly predict Fast-Growing AAAs with 100% accuracy, and the black dashed line is the null predictor. (**b**) Order of the most important features used in finalized models.

Models 8 to 12 evaluated the role of WSS, flow vortices, WSS-informatics, and velocity-informatics in predicting the AAAs. As indicated in Models 8 and 9, WSS and flow vortices were added to Model 6; AUROC decreased by 1% in both models. Also, those modifications decreased the total accuracy for Model 8 and Model 9 by 0.7% and 2.4%, respectively. In Model 10, WSS-informatics was added to Model 6, improving the AUROC and the total accuracy by 2% and 3%, respectively. In Model 11, velocity-informatics parameters were added to Model 7, and we found our most promising result. By involving these new features, AUROC and accuracy improved to 0.93% and 87.83%, with 82.00% and 90.75% fast- and slowly-growing identification. Finally, Model 12 comprises Model 11 and WSS-informatics, leading to 0.93 mean AUROC, 83.10% total accuracy, and 72% and 88.75% fast- and slowly-growing AAAs, respectively. The receiving operator characteristics (ROC) curve and area under the curve (AUC) of the top 3 models and Model 7 (the baseline of the top three models) and the most important features used in these models can be seen in Fig. 6. In the finalized optimal model, surface area_t and GrayLevelNonuniformity.Dvelocity were the top two most important features. In this study, the subscript *t* indicates the surface area of ILT.

Table 7 presents the effectiveness of velocity-informatics in improving SVM with linear kernels and five other commonly used machine learning methods (SVM with polynomial kernels, Lasso, KNN, RF, GBM).

**Table 7:** Comparison between the performance of 6 different classification methods using the same input parameters.

Classifier	Model	Mean AUC (95%CI)	Total accuracy (%)	Fast-growing identified (%)	Slowly-growing identified (%)
SVM Liner	Model 7	0.86 (0.83–0.89)	78.50	58.00	88.75
	Model 11	0.93 (0.90–0.95) **(+ 7%)**	87.83 **(+ 10.33%)**	82.00 **(24%)**	90.75** (+ 2.00%)**
SVM Poly	Model 7	0.69 (0.67–0.73)	65.50	50.00	73.25
	Model 11	0.85 (0.83–0.86) **(+ 16%)**	81.16 **(+ 15.66%)**	82.50 **(32.50%)**	80.00 **(+ 6.75%)**
Lasso	Model 7	0.80 (0.77–0.81)	69.00	20.00	93.50
	Model 11	0.88 (0.86–0.90) **(+ 8%)**	73.00 **(+ 4.00%)**	32.00 **(12%)**	93.50 **(0.00%)**
KNN	Model 7	0.69 (0.66–0.71)	66.10	39.00	79.50
	Model 11	0.79 (0.78–0.82) **(+ 10%)**	77.67 **(11.57%)**	49.50 **(10.50%)**	91.75 **(+ 12.25%)**
RF	Model 7	0.76 (0.74–0.78)	73.10	32.50	93.50
	Model 11	0.80 (0.78–0.83) **(+ 4%)**	77.16 **(4.06%)**	48.50 **(16%)**	91.50 *(− 2.00%)*
GBM	Model 7	0.71 (0.68–0.74)	68.00	32.50	85.75
	Model11	0.74 (0.710.79) **(+ 3%)**	72.50 **(4.50%)**	42.00 **(9.50%)**	86.25 **(0.50%)**

Input parameters to Models 7 and 11 are shown in Table 5.

The numbers in bold and italics represent the increase and decrease percentages.

*GBM* gradient boosting model, *KNN* K-nearest neighbor, *RF* random forest, *SVM* support vector machine.

The performance of the SVM method (with linear kernels) was strong in both Model 7 and Model 11. In Model 7, the SVM Linear achieved a mean AUC of 0.86 and a total accuracy of 78.50%. However, it encountered difficulties in accurately identifying fast-growing AAAs, resulting in a relatively low success rate of 58.00%. Conversely, the SVM method with linear kernels demonstrated high proficiency in identifying slowly-growing AAAs, achieving an identification rate of 88.75%. Remarkable improvements were observed in Model 11 for the SVM Linear method. It exhibited an increased mean AUROC of 0.93 (+ 7%) and a higher total accuracy of 87.83 (+ 10.33%). Importantly, it displayed enhanced capability in correctly identifying fast-growing AAAs, with an 82.00 (+ 24%) identification rate. Furthermore, it maintained a high accuracy in identifying slowly-growing AAAs, achieving an identification rate of 90.75 (+ 2.00%).

Regarding the SVM Polynomial method, Model 7 resulted in a mean AUC of 0.69 and a total accuracy of 65.50%. Similar to the SVM Linear approach, it faced challenges in accurately identifying fast-growing AAAs, with a recognition rate of only 50.00%. However, it performed better in identifying slowly-growing AAAs, achieving an identification rate of 73.25%. In Model 11, the SVM Polynomial method also demonstrated notable improvements, with an increased mean AUC of 0.85 (+ 16%) and a higher total accuracy of 81.16 (+ 15.66%). These enhancements translate into improved identification rates for fast-growing (82.50, + 32.50%) and slowly-growing AAAs (80.00, + 6.75%).

The Lasso method (Model 7) exhibited moderate performance, with a mean AUC of 0.80 and a total accuracy of 69.00%. Again, it faced challenges in accurately identifying fast-growing AAAs (20.00%), but it demonstrated proficiency in identifying slowly-growing AAAs (93.50%). Model 11 showed improvements, with an increased mean AUC of 0.88 (+ 8%) and a slightly higher total accuracy of 73.00 (+ 4.00%). Notably, Model 11 demonstrated enhanced capability in correctly identifying fast-growing AAAs (32.00, + 12%), while maintaining high accuracy in identifying slowly-growing AAAs (93.50%).

Similarly, Model 7 of the KNN method achieved a mean AUC of 0.69 and a total accuracy of 66.10%. It was challenging to identify both fast-growing (39.00%) and slowly-growing AAAs (79.50%). However, Model 11 again showed significant improvements, with an increased mean AUC of 0.79 (+ 10%) and a higher total accuracy of 77.67 (+ 11.57%). These enhancements translate into improved identification rates for both fast-growing (49.50, + 10.50%) and slowly-growing AAAs (91.75, + 12.25%).

The RF method (Model 7) demonstrated moderate performance, with a mean AUC of 0.76 and a total accuracy of 73.10%. However, it could accurately identify only a limited number of fast-growing AAAs (32.50%). In contrast, it performed well in identifying slowly-growing AAAs (93.50%). Model 11 exhibited slight improvement, with an increased mean AUC of 0.80 (+ 4%) and a slightly higher total accuracy of 77.16 (+ 4.06%). Improvements in identifying fast-growing AAAs (48.50%, + 16%) were notable. However, the identification of slowly-growing AAAs slightly decreased (91.50%, − 2.00%).

Model 7 of the GBM method demonstrated moderate performance, with a mean AUC of 0.71 and a total accuracy of 68.00%. Its ability to identify fast-growing AAAs was low (32.50%), but it performed well in identifying slowly-growing AAAs (85.75%). Model 11 under GBM showed a marginal improvement, with an increased mean AUC of 0.74 (+ 3%) and a slightly higher total accuracy of 72.50 (+ 4.50%). It also showed slight improvements in correctly identifying fast-growing AAAs (42.00, + 9.50%) while maintaining similar accuracy in identifying slowly-growing AAAs (86.25, + 0.50%).

Collectively, as shown in Table 7, additional velocity-informatics parameters improved nearly all models’ performance (Model 7 vs. Model 11), increasing the AUROC and mean total accuracy by 6.84% and 8.38% on average, respectively. Furthermore, the inclusion of velocity-informatics parameters significantly improved the prediction accuracy for both fast- and slowly-growing AAAs, on average, by 17.41% and 3.25%, respectively.

## Discussion

The inclusion of velocity-informatics and WSS-informatics into our finalized model showed a remarkable improvement in prediction performances compared with recently published studies^12,25–29^ attempting to predict AAAs’ growth status (i.e., fast-growing versus slowly-growing). Recall that the range of AUROC in those five studies was between 0.79 and 0.86. Meyrignac et al.^29^ performed a similar analysis, i.e., combining geometrical characteristics of AAAs' lumen and WSS extrema with patient health information (PHI) to differentiate fast- and slowly-growing aneurysms. Their predictive model was comparable to Model 6 in Table 5, and their model's AUROC was 0.79. In contrast, our recent predictive modeling study^12^ included ILT and flow vortex analysis. As a result, with the inclusion of ILT, the AUROC in our recent study reached 0.86 (i.e., Model 7 in Table 5). Zhu et al.^52^ reported that the presence of ILT positively correlated with AAAs’ growth rates. Comparing Model 6 with Model 7 in Table 6, there was a slight improvement in AUROC. Our best model (Model 11) reached 0.93 in this study for two reasons. First, in Model 11, we also counted the ILT. Second, the spatial pattern features in velocity (i.e., velocity-informatics) played a more significant role. Our results showed a more sizable increase of AUC (by 0.07) when velocity-informatics features were added to our predictive model (Model 11 vs. Model 6). This conclusion was verified using five other machine-learning methods (see Table 7).

As shown in Fig. 6a, after the inclusion of velocity-informatics and WSS-informatics, SVM can identify nearly 100% of fast-growing AAAs with a 60% false positive rate (see the dashed red curve in Fig. 6a). Identifying AAAs with a high growth rate indicating a high probability of rupture under a reasonable false positive rate might be acceptable. At the same time, ongoing research efforts continue to reduce the false positive rate.

**Figure 7:**
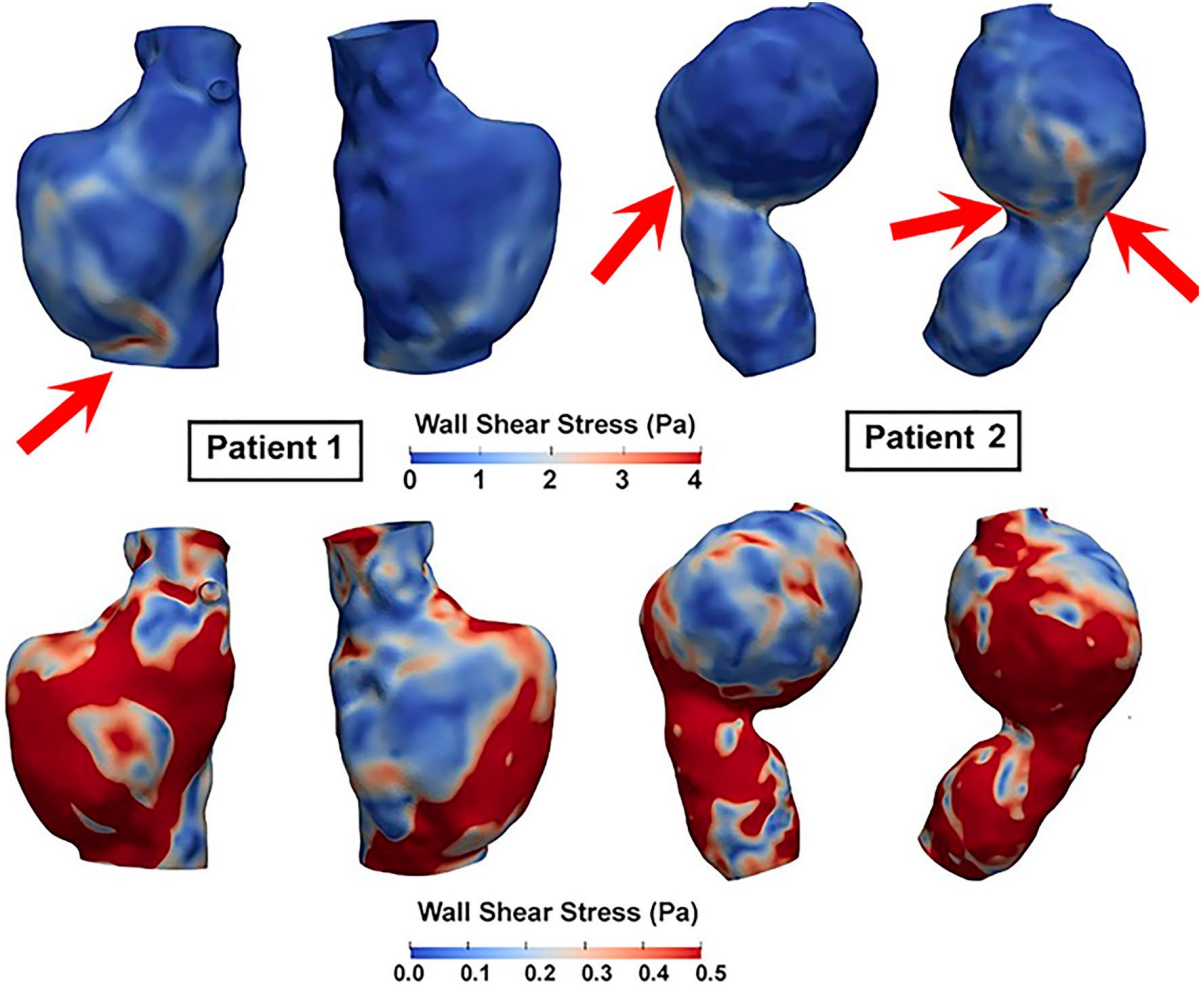
WSS Distributions in two AAAs at the peak systole. WSS values in the top row are scaled to visualize high WSS, while images in the second row are scaled to visualize low WSS distribution (0–0.5 Pa). Red arrows point to relatively high WSS.

**Figure 8:**
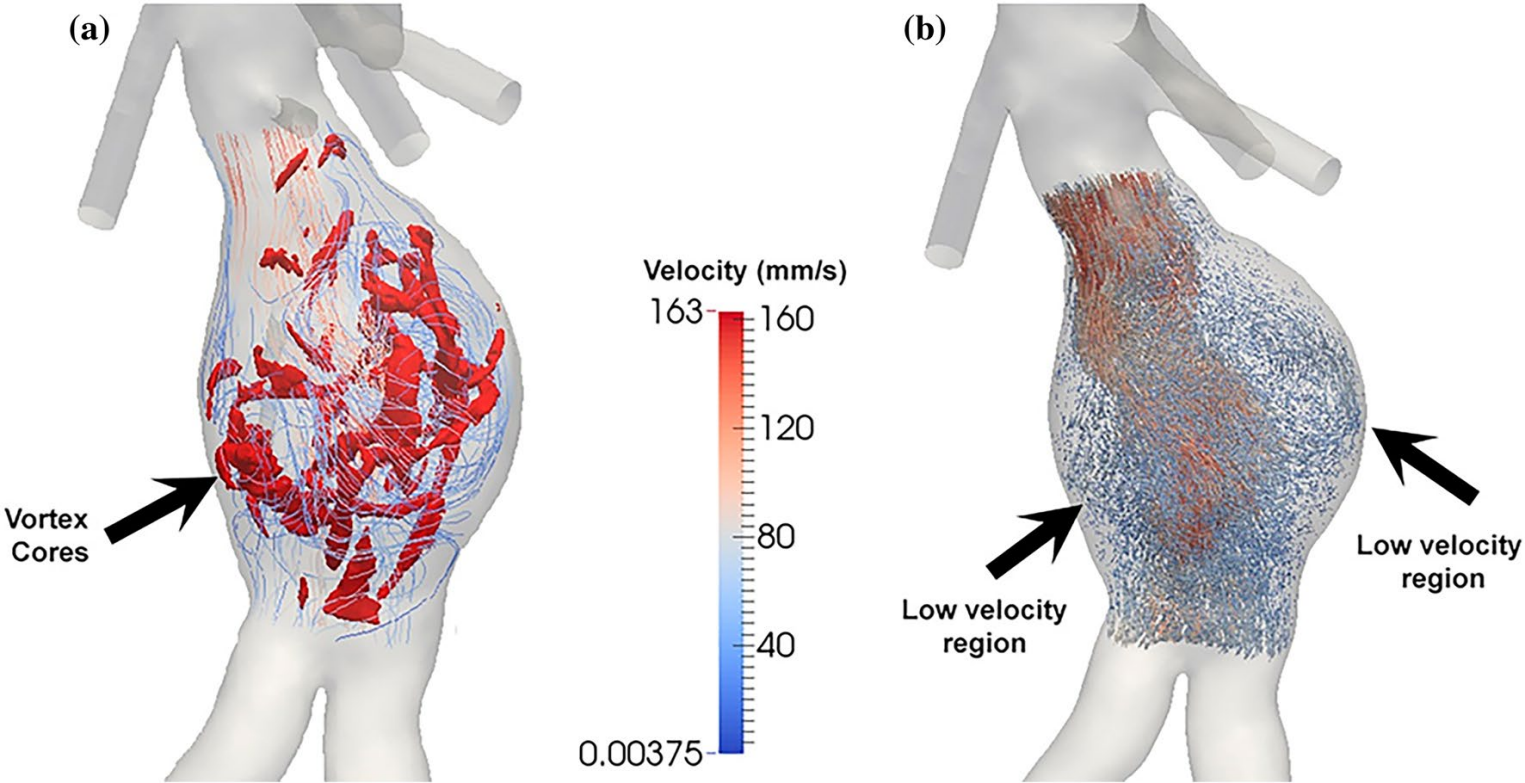
An example of disturbed flow within AAA: (**a**) streamlines to illustrate the position of (flow) vortice cores and (**b**) pathline to visualize recirculation region(s) and stagnation zone(s). Blue arrows in (**b**) refer to stasis inside the AAA, whereas the arrow in (**a**) shows vortices cores regions.

WSS-informatics and velocity-informatics improved the performance of predicting AAAs' growth status because they can better quantify the aneurismal flow disturbance, whereas conventional WSS parameters are not as effective. This observation is demonstrated using two showcase examples below in Figs. 7 and 8.

As shown in Table 6 (Model 8 versus Model 7), adding conventional WSS variables did not improve the prediction results. This observation underscores the limitation of how WSS is quantified, i.e., WSS extrema and Oscillatory Shear Index that only provide us with basic information about the magnitude and change of WSS vector directions^29,53,54^. Figure 7 shows an example of the WSS Minimum, Maximum, and average WSS within two AAAs. Obviously, as depicted in Fig. 7 (two images on the top row), those two cases have two different WSS distributions. For example, as highlighted with the red arrows in Fig. 7, a small portion of AAA1 is exposed to high WSS (> 3 Pa), but more areas of AAA2 are exposed to high WSS (> 3 Pa). Also, as illustrated in the bottom row of Fig. 7, showing WSS distribution above 0.5 Pa, regions with medium WSS exposure (between 0.5 and 3 Pa) are larger in one AAA (AAA1) compared with another AAA (AAA2).

In contrast, our study found that LargeArealowGraylevelEmphasis (LALGLE) of WSS directions (DWSS) can improve our ability to differentiate AAAs’ growth status. LALGLE can be evaluated as follows,

1 $$\mathrm{LALGLE}.\mathrm{DWSS}=\frac{\sum_{\mathrm{i}=1}^{{\mathrm{N}}_{\mathrm{g}}}\sum_{\mathrm{j}=1}^{{\mathrm{N}}_{\mathrm{s}}}\frac{ {DWSS}_{ij}^{2}}{{\mathrm{i}}^{2}}}{{\mathrm{N}}_{\mathrm{z}}}$$

where N_s_, N_g,_ and N_z_ represent the number of discrete zone sizes. In this context, high and low gray levels in DWSS denote downward (i.e., from the aorta to the iliac) and upward (i.e., from the iliac to the aorta) WSS vectors, respectively. As shown in Table 3, in slowly-growing AAAs, the LALGLE values of DWSS were larger than those in fast-growing AAAs (P-value = 0.11). Because the blood flows from the aorta to the iliac and more WSS vectors are aligned with the (downward) flow-driven direction, the presence of more upward WSS vectors (i.e., high LALGLE values of DWSS) is an indication of recirculation zones near the AAA wall. Consequently, quantifying LALGLE values of DWSS brought new information that is otherwise unavailable.

Similarly, adding flow vortex core information did not improve the prediction results (Model 9 versus Model 7 in Table 6). As shown in Fig. 8, complex flow distributions, e.g., vortices, stagnation zone, and recirculation regions, are often observed in AAAs. Compared with flow vortex core analysis (e.g., the number of cores, volume overlap, etc.), the proposed spatial pattern analysis of velocity provides more comprehensive information. For instance, GraylevelNonUniformity (GLN) of velocity directions (DVel) is defined as follows,

2 $$\mathrm{GLN}.\mathrm{DVel}=\frac{\sum_{\mathrm{i}=1}^{{\mathrm{N}}_{\mathrm{g}}}{\left(\sum_{\mathrm{j}=1}^{{\mathrm{N}}_{\mathrm{s}}}\mathrm{DVel}\left(\mathrm{i},\mathrm{j}\right)\right)}^{2}}{{\mathrm{N}}_{\mathrm{z}}}$$

where N_s_, N_g,_ and N_z_ represent the number of discrete zone sizes. A lower GLN value correlates with a greater similarity in the velocity directions, whereas a higher GLN value indicates greater variations in the velocity directions. Table 4 shows variations in velocity directions (GLN.DVel; P-value = 0.08) were greater in slowly-growing AAAs. Again, this result suggests that slowly-growing AAAs may have larger recirculation zones and other regions showing greater velocity direction variations within their sacs. In contrast, flow vortex core analysis (Fig. 8a) also quantifies rotational flow within the AAAs. But, it appears that GLN.DVel is more sensitive to the velocity's directional variations and can better quantify aneurismal flow disturbance.

Overall, the presence of a more significant degree of recirculation zones in slowly-growing AAAs, as indicated by LALGLE.DWSS and GLN.DVel, implies a higher degree of flow stagnation. This inference is consistent with slowly-growing AAAs containing more ILT (see Table 2). Although this inference is not surprising, data-driven discovery of this kind is still new, stimulating new research.

In summary, WSS-informatics and velocity-informatics show promise for predicting the growth status of AAAs in this feasibility study. This study has several limitations. First, the study included only a low number of AAAs. Our ongoing research is to validate our findings with a large cohort of patients. It is anticipated that an increased sample size will confirm data stability. Second, WSS-informatics and velocity-informatics in this study were computed at the peak systole instead of the entire cardiac cycle. The initial selection of this phase was motivated by the fact that blood attained its highest velocity during this period, leading to the manifestation of a relatively high degree of flow disturbance. We are in the process of considering time-resolved information from WSS-informatics and velocity-informatics. As part of our future plan, we intend to expand our analysis to include other phases during the cardiac cycle. Time-resolved hemodynamics-informatics will enable us to investigate the temporal stability of blood flow, gaining a deeper understanding of the temporal dynamics of flow characteristics and their relevance to AAAs’ progression. Third, CFD simulation models were based on a rigid wall assumption, which could affect hemodynamics near-wall. Also, “patient-specific” blood flow rate waveforms may further enhance the accuracy of our computed aneurismal hemodynamics. Although this effect may not have a critical impact on results, we will improve the realism of our hemodynamic analytics in the future, e.g., considering a compliant arterial wall and introducing “patient-specific” flow waveforms.

In summary, WSS-informatics and velocity-informatics show promise for predicting the growth status of AAAs in this feasibility study. Our ongoing research is to (1) validate our findings with a large cohort of patients with AAAs and (2) develop more sophisticated analytics to include transient spatial patterns in hemodynamics data. The rigor of our methodology can be enhanced if our observed spatial patterns in hemodynamics data can be connected to underlying vascular remodeling.

### Supplementary Information

Supplementary Information 1.

Supplementary Information 2.

## Data Availability

Imaging data were acquired at Mayo Clinic (Rochester, MN, USA). Derived data supporting this study will be made available from the corresponding author (JJ) upon request.
