# Supplementary material for: Characterization of small abdominal aortic aneurysms' growth status using spatial pattern analysis of aneurismal hemodynamics
Source: Sci Rep. 2023 Aug 24;13:13832. doi: 10.1038/s41598-023-40139-z (PMC10449842; doi:10.1038/s41598-023-40139-z)
Supplement: Supplementary file 1 — Supplementary Information 1. [file 41598_2023_40139_MOESM1_ESM.pdf]

Characterization of Small Abdominal Aortic Aneurysms' Growth Status Using Spatial Pattern Analysis of Aneurismal Hemodynamics

Mostafa Rezaeitaleshmahalleh [1,2], Zonghan Lyu [1,2], Nan Mu [1,2], Xiaoming Zhang [3], Todd E. Rasmussen [4], Robert D. McBane II [5] and Jingfeng Jiang [1,2,3,*]

[1]Dept. of Biomedical Engineering, Michigan Technological University, Houghton, Michigan
[2]Joint Center for Biocomputing and Digital Health, Health Research Institute, and Institute of Computing and Cybernetics, Michigan Technological University, Houghton, Michigan
[3]Dept. of Radiology, Mayo Clinic, Rochester, Minnesota
[4] Division of Vascular and Endovascular Surgery, Mayo Clinic, Rochester, Minnesota
[5]Dept. of Cardiovascular Medicine, Mayo Clinic, Rochester, Minnesota
[*]Corresponding author: jjiang1@mtu.edu

Appendix: Definitions of Textural Features

A. Intensity-based Hemodynamics-informatics Features

The descriptions below are similar to that provided in PyRadiomics documentation and are provided below for completeness.

A.1 First-Order Statistics

First-order statistics features represent the distribution of distinct voxel values without considering the inter-relationship with neighboring voxels. These histogram-based properties identify the mean, median, maximum, and minimum values of the voxel intensities on the image-like hemodynamic data and their asymmetry, flatness, uniformity, and entropy.

A.2 Second-Order Statistics:

In contrast, second-order statistics is related to information regarding the inter-relationship between neighboring voxel intensities and their spatial arrangement. Several methods that can quantify the voxels' inter-relationship have been reported, namely, Gray level co-occurrence matrix (GLCM)[1], Gray level run length matrix (GLRLM)[2] , and Gray level size zone matrix (GLSZM)[3]. Parameters derived from GLCM, GLRLM, and GLSZM are summarized below.

To better comprehend voxels' inter-relationship analysis, graphical examples are provided below. In the examples below, we use images with discrete intensity values ranging from 1 to 5, and, as a result, each intensity value corresponds to one unique color.

A.2.1 Gray level co-occurrence matrix (GLCM)

Gray level co-occurrence matrix (GLCM) is computed based on the relationship between two connected voxels and the commonness of this connection and defined as $P(i, j|\delta, \theta)$. This relationship is represented using two components, distance (δ) and angle between two voxels θ, as shown in Fig. A.1(a). θ is one of four discrete values for 2D images, and this number increase to 13 for 3D images. Mathematically, the GLCM of an image (or image-like data) with Nx ×Ny dimensions and Ng different intensity levels is computed using the following equation[1,4]:

$$\mathrm{GLCM}_{\delta}^{\theta}(i, j)=\left|\left\{\left((r,s),(t,v)\right): \mathbf{I}(r,s)=i,\ \mathbf{I}(t,v)=j\right\}\right| \ \forall\, i,j \in \{1,2,3,\dots,N_g\} \tag{A-1}$$

Where $(t,v)=\begin{cases} r+\delta, s & if\ \theta=0^{o} \\ r+\delta, s+\delta & if\ \theta=45^{o} \\ r, s+\delta & if\ \theta=90^{o} \\ r-\delta, s+\delta & if\ \theta=135^{o} \end{cases}$ and $|.|$ represents the number of components in a set.

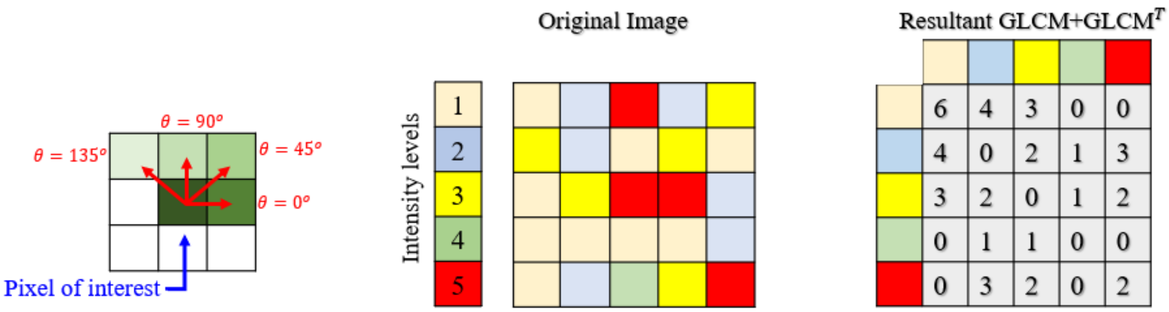

Figure A.1: An illustration of GLCM extraction. (a) An illustration of user-defined parameter, θ in this process between two connected pixels (b) Original image (b) Resultant GLCM calculated by adding asymmetric GLCM with its transpose.

Fig. A.1 depicts an example of a 5 ×5 matrix with the related GLCM for θ=0 and δ =1. It should be considered that different value θ and δ can produce different GLCM. By default, the value of features is calculated separately for any angle, and finally, a mean value of calculated features is returned.

For each GLCM, twenty-four features were developed. Top features in this study are described as follows and can be directly calculated using the open-source python PyRadiomics1 package.

Autocorrelation: Quantifies the magnitude of fineness and coarseness of an image texture and can be estimated as:

$$\text{Autocorrelation}=\sum_{i=1}^{N_g}\sum_{j=1}^{N_g} \boldsymbol{p(i,j)i} \quad \text{(A-2)}$$

Cluster Shade: Quantifies the level of skewness and uniformity of GLCM, and its higher value implies more significant asymmetry around the average value.

$$\text{Cluster Shade} =\sum_{i=1}^{N_g}\sum_{j=1}^{N_g}(i+j-\mu_x-\mu_y)^3 p(i,j) \quad \text{(A-3)}$$

Cluster Tendency: Measures sets of voxels with an identical intensity value.

$$\text{Cluster Tendency}=\sum_{i=1}^{N_g}\sum_{j=1}^{N_g}(i+j-\mu_x-\mu_y)^2 p(i,j) \quad \text{(A-4)}$$

Joint Average: Compute mean intensity level *i* distribution.

$$\text{Joint Average}=\mu_x=\sum_{i=1}^{N_g}\sum_{i=1}^{N_g} p(i,j)i \quad \text{(A-5)}$$

Joint Entropy: Quantifies the range of randomness in adjacent intensity values.

$$\text{Joint Entropy} =-\sum_{i=1}^{N_g}\sum_{j=1}^{N_g} p(i,j)\log_2(p(i,j)+\varepsilon) \quad \text{(A-6)}$$

Joint Energy: Estimates the homogeneity of a pattern in the image.

$$\text{Joint Energy} = \sum_{i=1}^{N_g}\sum_{j=1}^{N_g}(p(i,j))^2 \qquad \text{(A-7)}$$

Maximum Probability: Quantifies the number of the most common set of adjacent intensity levels.

$$\text{Maximum Probability} = \max(p(i,j)) \qquad \text{(A-8)}$$

Sum Average: Estimates the association between the occurrences of connected pixels with lower and higher intensity levels.

$$\text{Sum Average} = \sum_{\kappa=2}^{2N_g} P_{x+y}(\kappa)\kappa \qquad \text{(A-9)}$$

Here, $P_{x+y}(\kappa) = \sum_{i=1}^{N_g}\sum_{j=1}^{N_g} p(i,j)$, where i+j= κ and κ =2,3, …, $2N_g$

Sum Entropy: Aggregates of neighborhood intensity values distinctions.

$$\text{Sum Entropy} = \sum_{i=1}^{N_g}\sum_{j=1}^{N_g} P_{x+y}(\kappa)\log_2(P_{x+y}(\kappa) + \varepsilon) \qquad \text{(A-10)}$$

A.2.2 Gray level run length matrix (GLRLM)

Gray level run length matrix (GLRLM)[2] is computed based on the number of connected voxels in the same intensity. GLRLM is characterized by an angle between pairs of voxels, θ. Elements (i,j) in the matrix represents the number of voxels with intensity i and run length j in a specified direction.

Mathematically, the GLRLM of an image with Nx ×Ny dimensions and Ng different intensity levels is computed using the following equation[2,4]:

$$GLRLM_\theta(i,j) = |\{(m,n): |\{(k,l)\epsilon\ Nb(m,n,j,\theta): I(k,l) = i\}|\ \forall\ \mathrm{i,j} \in \{1,2,3,\dots,\mathrm{N_g}\} \qquad \text{(A-11)}$$

Where $Nb(m,n,j,\theta) = \begin{cases} \{(m+1,n),(m+2,n),\dots,(m+j,n)\} & if\ \theta = 0^o \\ \{(m+1,n+1),(m+2,n+2),\dots,(m+j,n+j)\} & if\ \theta = 45^o \\ \{(m,n+1),(m,n+2),\dots,(m,n+j)\} & if\ \theta = 90^o \\ \{(m-1,n-1),(m-2,n-2),\dots,(m-j,n-j)\} & if\ \theta = 135^o \end{cases}$ and $|.|$

denotes the number of elements in a set.

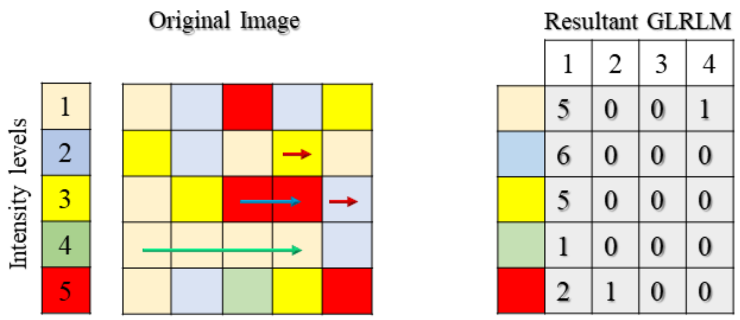

Figure A.2: An illustration of GLRLM extraction. (a) Original image (b) Resultant $GLRLM_{\theta=0}$. Red, blue, and green arrows represent pixels in the same intensity level along $\theta = 0$ with Run Length=1,2 and 4, respectively.

Fig. A.2 depicts an example of a 5 ×5 matrix with the related GLRLM for θ=0. Different θ value can produce different GLRLM. For each GLRLM, sixteen features have been developed. Top features are summarized below, and all of them can be calculated using open-source PyRadiomics packages:

HighGrayLevelRunEmphasis: Quantifies the distribution of voxels with higher-intensity values.

$$\text{HighGrayLevelRunEmphasis} = \frac{\sum_{i=1}^{N_g}\sum_{j=1}^{N_r} P(i,j|\theta) i^2}{N_r(\theta)} \qquad \text{(A-12)}$$

$N_r(\theta)$ is a number of runs in an image along angle θ and calculated as follows:

$$N_r(\theta) = \sum_{i=1}^{N_g}\sum_{j=1}^{N_r} \boldsymbol{P}(i,j|\theta) \ , \qquad 1 \le N_r(\theta) \le N_p$$

LongRunHighGrayLevelEmphasis: Quantifies joint distribution of voxels with higher intensity and long run length.

$$\text{LongRunHighGrayLevelEmphasis} = \frac{\sum_{i=1}^{N_g}\sum_{j=1}^{N_r} P(i,j|\theta) i^2 j^2}{N_r(\theta)} \qquad \text{(A-13)}$$

LongRunLowGraylevelEmphasis: Quantifies joint distribution of voxels with lower intensity and long run length.

$$\text{LongRunLowGraylevelEmphasis} = \frac{\sum_{i=1}^{N_g}\sum_{j=1}^{N_r} \frac{P(i,j|\theta) j^2}{i^2}}{N_r(\theta)} \qquad \text{(A-14)}$$

LowGrayLevelRunEmphasis: estimate the distribution of images' lower intensity values. .

$$\text{LowGrayLevelRunEmphasis} = \frac{\sum_{i=1}^{N_g}\sum_{j=1}^{N_r} \frac{P(i,j|\theta)}{i^2}}{N_r(\theta)} \qquad \text{(A-15)}$$

RunVariance: **Measures** variance of runs based on existing run length.

$$\text{RunVariance} = \sum_{i=1}^{N_g} \sum_{j=1}^{N_r} P(i,j|\theta)\,(j-\mu)^2 \quad \text{(A-16)}$$

Here, $\mu = \sum_{i=1}^{N_g} \sum_{j=1}^{N_r} P(i,j|\theta)\, j$

A.2.3 Gray level size zone matrix (GLSZM)

Gray level size zone matrix (GLSZM) was first defined by Thibault et al[3] to quantify intensity zones in an image. A zone is defined as some connected voxels with the same intensity level. The (i,j)th element of GLSZM represents the number of zones with intensity i and size j seen in the image. Unlike the GLCM and GLRLM, there is no dependency on the θ to the generation of GLSZM, and thus, only a unique matrix will be calculated considering different directions.

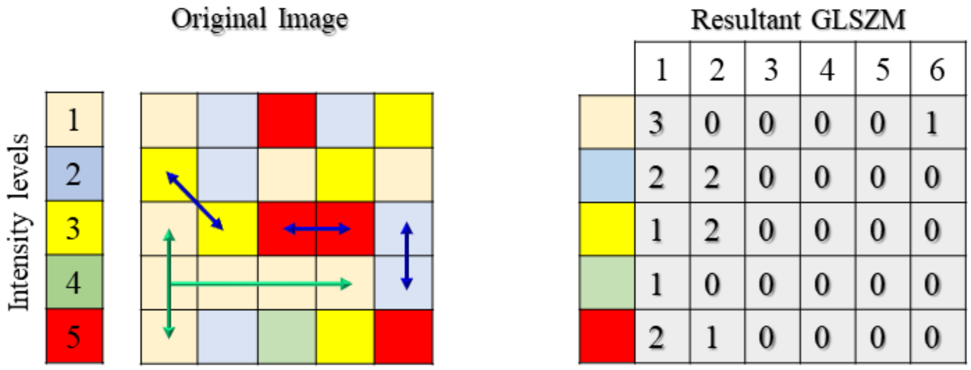

Figure *A.3*: An illustration of GLSZM extraction. (a) Original image (b) Resultant GLSZM. Blue and green arrows represent pixels in the same intensity level with size zone=2 and 6, respectively.

Fig. A.3 illustrates an example of a 5 ×5 matrix with the related GLSZM. For each GLSZM, sixteen features can also be calculated using the open-source PyRadiomics package. We explain the top-ranked features below:

HighGraylevelzoneEmphasis: Measures the distribution of higher intensity size zones.

$$\text{HighGraylevelzoneEmphasis} = \frac{\sum_{i=1}^{N_g} \sum_{j=1}^{N_s} P(i,j) i^2}{N_z} \quad \text{(A-17)}$$

GrayLevelVariance: Measures variance of intensity level based on existing zones.

$$\text{GrayLevelVariance} = \sum_{i=1}^{N_g} \sum_{j=1}^{N_s} P(i,j|\theta)(j-\mu)^2 \quad \text{(A-18)}$$

Here, $\mu = \sum_{i=1}^{N_g} \sum_{j=1}^{N_s} P(i,j|\theta)\, j$

Zone Entropy: Quantifies the texture's coarseness based on the ratio between number of zones and number of voxels.

$$\text{Zone Entropy} = \frac{N_z}{N_P} \tag{A-19}$$

Here, N_z represents the number of zones in ROI and is calculated as follows:

$$N_z = \sum_{i=1}^{N_g} \sum_{j=1}^{N_r} \boldsymbol{P}(i,j)$$

A.3 Higher-order statistics

Higher-order statistics features are computed using statistical methods after utilizing a filter or mathematical transform on the original image. The primary purpose of applying these techniques is to cancel noise, highlight detail, and identify repetitive and non-repetitive patterns. Including current existing techniques, we implemented the LoG filter, which can increasingly highlight coarse texture patterns and wavelet transform (WT) analyses image texture at different levels.

A.3.1 Laplacian of Gaussian (LoG) Filter

The Laplacian filter applies to the image to detect rapid changes in intensity levels, but this procedure is susceptible to noise. Therefore, the Gaussian filter is first applied to obtain a smooth image before the Laplacian filter. The above-mentioned two combined steps are known as the Laplacian of Gaussian (LoG) filter and aim at reducing the image noise level, improving the measurement of image heterogeneity[5].

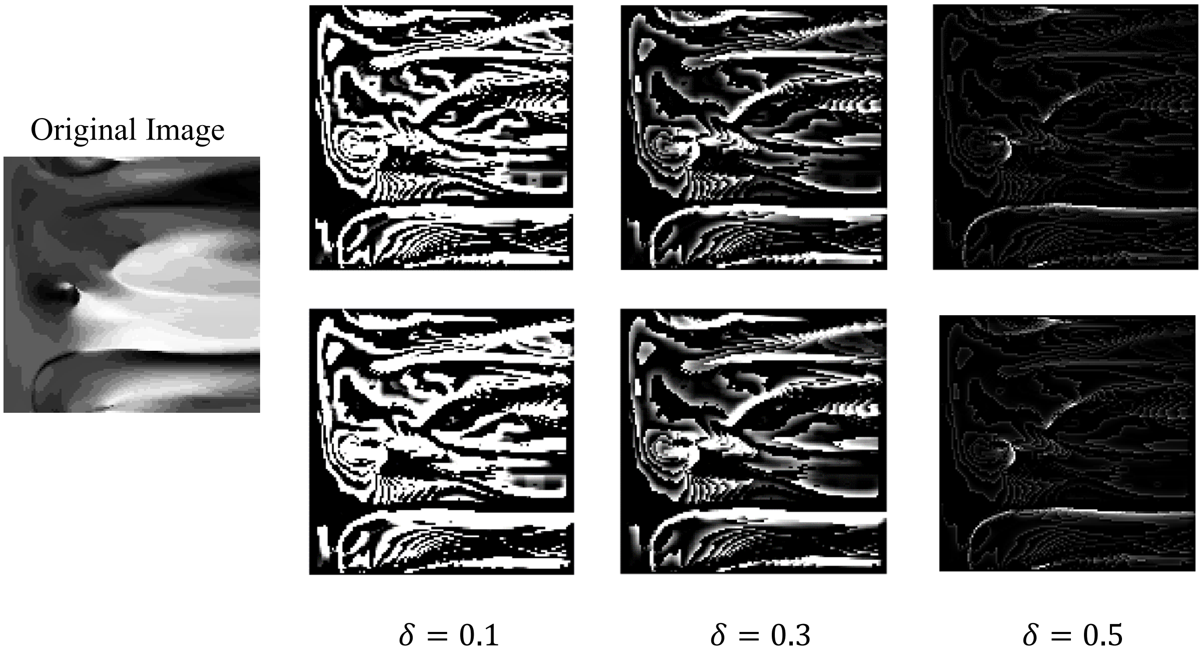

Figure A.4: An illustrative example showing the effects of LoG. The first column is related to an (original) image from DWSS-informatics. Columns 2-4 are resultant images post-LoG filters when δ is equal to 0.1,0.3, and 0.5, respectively.

A Gaussian kernel function is defined as follows:

$$G(x, y, z, \delta) = \frac{1}{(\delta\sqrt{2\pi})^3} e^{-\frac{x^2+y^2+z^2}{2\delta^2}} \quad \text{(A-20)}$$

Then, the Gaussian function is convolved by a Laplacian function to obtain a single equation, $\mathscr{L}(G(x, y, z)$. δ in Eq. (A-20) denotes the filter's width and correlates with the output image's texture level. So, low values highlight more fine textures, and higher values identify coarse textures (See Fig.A.4).

A.3.2 Wavelet transforms

Original Image WT is a mathematical technique to decompose original images into different sub-bands levels for generating multi-resolution images. Hence, WT can help to extract hidden information from an image[3,6-9]. More specifically, Haar, dmey, sym, db, coif, bior, and rbio are currently available wavelet types (PyWavelet version 0.4.0) and provide low-frequency and high-frequency information[10].

To start this process, one of these filter types is first applied to the original image on the x-axis. Then, one high-pass and one low-pass image are obtained, labeled as H and L, respectively. Then, this process repeats in the y-

axis and produces four sub-bands viz. HL(Vertical), HH(Diagonal), LH(Horizontal), and LL(Approximation). An example of this process is depicted in Fig. A.5, in which coif1 with level 1 is applied on a 2D DWSS image. A 3D image has one more step, and a WT needs to be applied to the z-axis on these four sub bands. As a result, we obtain HLL, HLH, HHH, HHL (high-pass along the z-axis) and LHL, LHH, LLH, LLL (loss-pass along the z-axis) images. By continually applying WT on LLL in 3D and LL in 2D images at each level, images with distinct resolution levels can be generated[4,11,12].

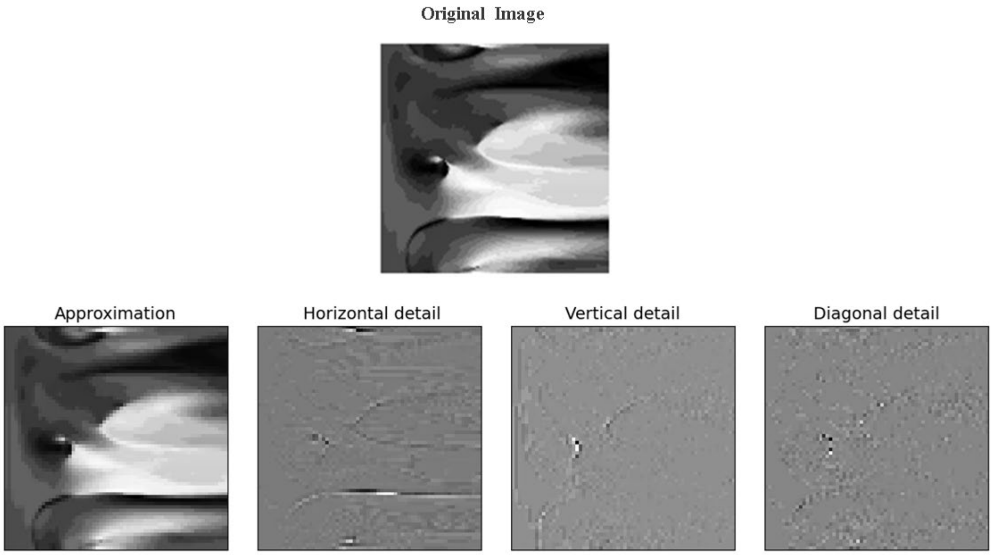

Figure A.5: An illustration of Wavelet transform outcomes using coif1 wavelet filter bank with one level of wavelet decomposition. In this case, the original image is a DWSS image.

References

1 Haralick, R. M., Shanmugam, K. & Dinstein, I. H. Textural features for image classification. *IEEE Transactions on systems, man, and cybernetics*, 610-621 (1973).

2 Galloway, M. M. Texture analysis using gray level run lengths. *Computer graphics and image processing* **4**, 172-179 (1975).

3 Thibault, G., Angulo, J. & Meyer, F. Advanced statistical matrices for texture characterization: application to cell classification. *IEEE Transactions on Biomedical Engineering* **61**, 630-637 (2013).

4 Parekh, V. & Jacobs, M. A. Radiomics: a new application from established techniques. *Expert review of precision medicine and drug development* **1**, 207-226 (2016).

5 Yasaka, K. *et al.* Precision of quantitative computed tomography texture analysis using image filtering: a phantom study for scanner variability. *Medicine* **96** (2017).

6 Daubechies, I. *Ten lectures on wavelets*. (SIAM, 1992).

7 Daubechies, I. Orthonormal bases of compactly supported wavelets. *Communications on pure and applied mathematics* **41**, 909-996 (1988).

8 Haar, A. *Zur theorie der orthogonalen funktionensysteme*. (Georg-August-Universitat, Gottingen., 1909).

9 Mallat, S. G. A theory for multiresolution signal decomposition: the wavelet representation. *IEEE transactions on pattern analysis and machine intelligence* **11**, 674-693 (1989).

10 Van Griethuysen, J. J. *et al.* Computational radiomics system to decode the radiographic phenotype. *Cancer research* **77**, e104-e107 (2017).

11 Chaddad, A., Daniel, P. & Niazi, T. Radiomics evaluation of histological heterogeneity using multiscale textures derived from 3D wavelet transformation of multispectral images. *Frontiers in oncology* **8**, 96 (2018).

12 Zhou, J. *et al.* Predicting the response to neoadjuvant chemotherapy for breast cancer: wavelet transforming radiomics in MRI. *BMC Cancer* **20**, 1-10 (2020).
