# Supplementary material for: Characterization of small abdominal aortic aneurysms' growth status using spatial pattern analysis of aneurismal hemodynamics
Source: Sci Rep. 2023 Aug 24;13:13832. doi: 10.1038/s41598-023-40139-z (PMC10449842; doi:10.1038/s41598-023-40139-z)
Supplement: Supplementary file 2 — Supplementary Information 2. [file 41598_2023_40139_MOESM2_ESM.pdf]

Characterization of Small Abdominal Aortic Aneurysms' Growth Status Using Spatial Pattern Analysis of Aneurismal Hemodynamics

Mostafa Rezaeitaleshmahalleh [1,2], Zonghan Lyu [1,2], Nan Mu [1,2], Xiaoming Zhang [3], Todd E. Rasmussen [4], Robert D. McBane II [5] and Jingfeng Jiang [1,2,3,*]

[1]Dept. of Biomedical Engineering, Michigan Technological University, Houghton, Michigan
[2]Joint Center for Biocomputing and Digital Health, Health Research Institute, and Institute of Computing and Cybernetics, Michigan Technological University, Houghton, Michigan
[3]Dept. of Radiology, Mayo Clinic, Rochester, Minnesota
[4] Division of Vascular and Endovascular Surgery, Mayo Clinic, Rochester, Minnesota
[5]Dept. of Cardiovascular Medicine, Mayo Clinic, Rochester, Minnesota
[*]Corresponding author: jjiang1@mtu.edu

Morphological Analysis

As depicted in Fig.S.1, to calculate the geometric features of these two regions, each AAA sac was isolated from its parent vessel (i.e., an STL file as mentioned above)[1]. The following AAA geometric characteristics were estimated: volume, surface area, length, mean AAA radius, max AAA radius, min AAA radius, parent vessel circumference (distal to AAA), parent vessel radius (proximal to AAA), expansion ratio (mean AAA radius/vessel radius proximal to AAA), aspect ratio ((length* parent vessel circumference (distal to AAA))/(4*Ostium area)), (parent vessel) inlet surface area, tortuosity and undulation index (UI).

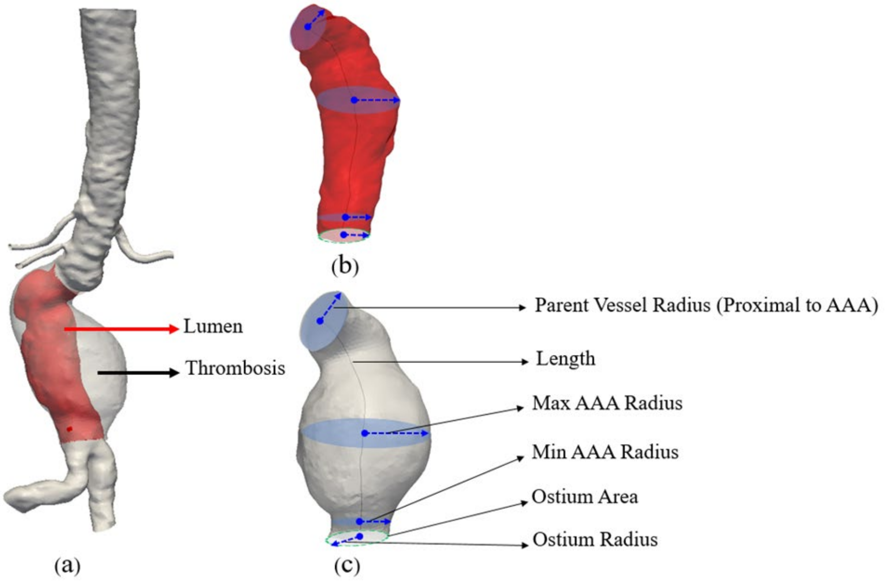

Figure.S.1: An illustration of geometric characteristics of typical AAAs: (a) an extended aorta model including both lumen (red) and intraluminal thrombosis (ILT; gray), (b) Surface of the lumen, (c) Surface of ILT. The solid black lines in (b) and (c) are the generated centerlines based on the Voronoi diagram. In (d), the blue circles are two 2D planes from which Min:Max Radius is measured, and the dashed green line in the bottom cut plan shows the ostium circumference.

Vessel tortuosity and UI were calculated following methods similar to Piccinelli et al[2]. and Dhar et al[3]., respectively. Both tortuosity and UI are available through the VMTK package.

In addition to the abovementioned indices, seven new geometric variables were defined based on dimensions of the lumen and ILT region to combine characteristics of these two regions as follows:

$$\text{NTTD}=\left(\frac{\text{max AAA radius\_t}-\text{min AAA radius\_t}}{\text{mean AAA radius\_t}}\right) \quad (1)$$

$$\text{NTT1}=\left(\frac{\frac{\text{max AAA radius\_t}-\text{min AAA radius\_t}}{\text{mean AAA radius\_t}}}{\frac{\text{max AAA radius}-\text{min AAA radius}}{\text{mean AAA radius}}}\right) \quad (2)$$

$$\text{NTT2}=\left(\frac{\text{max AAA radius\_t}-\text{mean AAA radius\_t}}{\text{mean AAA radius\_t}}\right) \quad (3)$$

$$\text{NTNU1}=\left(\frac{\text{max AAA radius\_t}-\text{min AAA radius\_t}}{\text{max AAA radius}-\text{min AAA radius}}\right) \quad (4)$$

$$\text{NTNU2}=\left(\frac{\text{max AAA radius\_t}\times\text{mean AAA radius}}{\text{max AAA radius}\times\text{mean AAA radius\_t}}\right) \quad (5)$$

$$\text{New\_Aspect\_Ratio\_t}=\left(\frac{\text{max AAA\_t radius}-\text{vessel radius proximal to AAA}}{Length\_t}\right) \quad (6)$$

$$\text{New\_Aspect\_Ratio}=\left(\frac{\text{max AAA radius}-\text{vessel radius proximal to AAA}}{Length}\right) \quad (7)$$

Hemodynamics Parameters

Wall Shear stress

Wall shear stress (WSS) is the consequence of the frictional/tangential forces on the endothelial surface of the blood vessel exerted by the blood flow and calculated as follows [4]:

$$\text{WSS}= \mu \dot{\gamma} \quad (8)$$

Where μ is the dynamics viscosity and $\dot{\gamma}$ is the share rate.

In summary, WSS was automatically calculated using a commercial CFD solver (Fluent, ANSYS Inc., USA). Then, the WSS's extrema and its spatial average value were computed based on the estimated WSS value in any timestep. Finally, the temporally averaged WSS Minimum, Maximum and spatial average values were calculated and called TA-WSS-Min, TA-WSS-Max, and STA-WSS, respectively.

Additionally, a nondimensional parameter Oscillatory Shear Index (OSI) was computed for each point on the vessel wall[5]:

$$OSI = 0.5\left(1-\frac{\left|\int_0^T \tau_i dt\right|}{\int_0^T |\tau_i| dt}\right) \quad (9)$$

where τ_i is a WSS vector located at a point at a given time step across the cardiac cycle duration (T). OSI represents the oscillations in WSS vector directionality over a cardiac cycle for a spatial location on the vessel wall: An OSI of 0 indicates no change in directionality, while 0.5 suggests a 180° angular change.

Degree of Volume Overlap

Calculating flow vortex-related parameters estimating based on core regions of swirling flow can be considered an appropriate method to show swirling flows' stability. To this end, a published method by our group[6,7] based on Shannon (informational) entropy was utilized to identify flow vortex. Both the swirling flow patterns and extracted central region flow vortices during the cardiac cycle vary, as shown in an illustrative example in Fig.S.2.

Tracking these changes can quantify **the temporal stability of flow vortices**. The degree of volume overlap (DVO) between identified vortices of adjacent time steps shows how swirling flow pattern configurations change in a specified time interval which lower DVO values indicating reduced temporal stability (See Fig.S.2). The first two metrics are temporally average DVO (TA-DVO) and DVO standard division (DVO-Std).

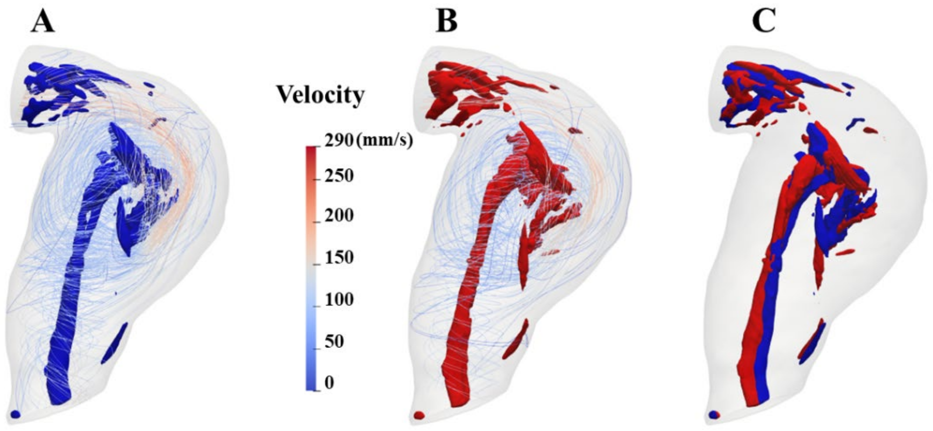

Figure.S.2: Examples of vortex iso-surfaces and their spatiotemporal variation between subsequent timesteps: (A) Timestep i iso-surfaces (blue), (B) timestep i+1 iso-surfaces (red), (C) both timesteps with iso-surface overlap. Velocity streamlines show the overall flow pattern and velocity (mm/sec) at each time step.

To highlight the spatial complexity of the aneurismal flow, the temporally-averaged number of identified vortical areas is considered an appropriate quantity in which a higher number indicates more flow complexity. In addition, VtV is another spatial metric defined as the total volume of the identified vortices core across a cardiac cycle normalized to the AAA's volume. Its higher number indicates that a high portion of the aneurysmal volume is occupied with swirling flow.

Mesh Sensitivity Analysis

To check the mesh sensitivity, we generated unstructured mesh for specific geometries with different mesh sizes. For instance, we chose one AAA model and meshed it with 2,4,7, and 10 million elements. Upon the completion of CFD simulations with different mesh sizes, we randomly selected 15 points approximately in the same location from all simulations and then calculated the Absolute Difference, Relative Difference, and Percent Difference of velocity's magnitude and pressure across four meshes.

In the first step, we compared CFD results using 2 and 4 million elements. As shown in Supplemental Table.S.1, there was a significant difference between velocity and pressure using 2 million elements, indicating a mesh with 2 million elements was too coarse. Then, we compared the outcomes of models with 4 and 7 million elements. As indicated in Supplemental Table.S.2, the models' differences were reduced comparatively but still relatively high (e.g., an 11% error in pressure). Finally, we compared CFD model results with 7 and 10 million elements. As shown in Supplemental Table.S.3, the results stayed mostly the same (less than 3% differences), indicating that we can trust a mesh size between 7 to 10 million elements. Given the long computing time for such a CFD simulation, we opted for a mesh size of around 7 million elements.

Table.S.1: Velocity magnitude and total pressure compared through absolute, relative, and percent differences in 2 million and 4 million element quantity meshes. The averages of each difference are displayed in the bottom row

**2 vs 4 Million Elements**					
**Absolute Difference**		**Relative Difference**		**Percent Difference**	
**Velocity**	**Pressure**	**Velocity**	**Velocity**	**Pressure**	**Velocity**
0.01805	12.34220	0.46019	-0.13461	59.77268	-12.61211
0.01059	12.07710	0.30622	-0.13345	36.15756	-12.51066
0.00927	11.48950	-0.41781	-0.12324	34.56109	-11.60846
0.00032	12.45800	-0.00881	-0.13453	0.87665	-12.60523
0.00835	11.77160	0.32686	-0.12778	39.07152	-12.01034
0.00264	10.46940	-0.20345	-0.11270	18.46671	-10.66920
0.00032	12.45800	-0.00881	-0.13453	0.87665	-12.60523
0.00350	11.97080	0.10565	-0.13014	11.15410	-12.21889
0.01122	12.61750	-0.12108	-0.13604	11.41667	-12.73733
0.01434	11.84990	0.41694	-0.12835	52.67493	-12.06096
0.00275	12.14980	0.09266	-0.13500	9.71658	-12.64681
0.00227	13.22980	-0.02245	-0.14052	2.22000	-13.12909
0.02810	15.31080	-1.24886	-0.16520	76.88001	-15.25989
0.00464	12.99570	0.05002	-0.13846	5.13022	-12.94945
0.01851	13.58330	-0.38329	-0.14223	32.16487	-13.2789
.01805	12.34220	0.46019	-0.13461	59.77268	-12.61211
0.01059	12.07710	0.30622	-0.13345	36.15756	-12.51066
0.00927	11.48950	-0.41781	-0.12324	34.56109	-11.60846
0.00899	12.45156	-0.04373	-0.13445	26.07602	-12.59350

Table.S.2: Mesh sensitivity calculation using 4 and 7 million elements with absolute, relative, and percent differences. The averaged values are in the bottom row

4 vs 7 Million Elements					
Absolute Difference		Relative Difference		Percent Difference	
Velocity	Pressure	Velocity	Velocity	Pressure	Velocity
0.0080	5.4640	0.3815	-0.0525	-0.0075	-10.0723
0.0068	6.5600	0.2843	-0.0639	-0.0064	-12.2464
0.0084	7.4340	0.2697	-0.0709	-0.0078	-13.6211
0.0215	5.4290	0.5829	-0.0516	-0.0200	-10.1962
0.0019	6.4030	0.1102	-0.0616	-0.0017	-12.1613
0.0103	7.4630	-0.6599	-0.0722	-0.0096	-14.2389
0.0217	5.4810	0.5876	-0.0521	-0.0201	-10.5870
0.0058	6.6880	0.1969	-0.0643	-0.0054	-13.0315
0.0039	7.6430	-0.0380	-0.0725	-0.0036	-14.6965
0.0086	5.7160	0.4326	-0.0548	-0.0081	-11.4444
0.0030	6.6220	0.1134	-0.0648	-0.0029	-13.5464
0.0069	7.0000	-0.0674	-0.0651	-0.0062	-13.6742
0.0202	2.4350	0.4006	-0.0225	-0.0185	-4.9987
0.0015	7.1420	0.0178	-0.0668	-0.0014	-14.2844
0.0025	3.5670	0.0379	-0.0327	-0.0022	-7.3056
**0.0087**	**6.0698**	**0.1767**	**-0.0579**	**-0.0081**	**-11.7403**

Table.S.3 ***:*** Mesh sensitivity calculation using 4 and 7 million elements with absolute, relative, and percent differences. The averaged values are in the bottom row

7 vs 10 Million Elements					
Absolute Difference		Relative Difference		Percent Difference	
Velocity	Pressure	Velocity	Velocity	Pressure	Velocity
2.9727	0.0520	-0.0343	-0.0001	3.4941	0.0108
4.9971	0.3000	-0.0648	-0.0006	6.7035	0.0624
0.0299	0.0180	-0.0003	0.0001	0.0378	0.0038
0.3820	0.1850	-0.0035	-0.0004	0.3538	0.0381
**2.0954**	**0.1387**	**-0.0257**	**-0.0003**	**2.6473**	**0.0288**

References

1 Jiang, J. & Strother, C. M. Interactive decomposition and mapping of saccular cerebral aneurysms using harmonic functions: its first application with "patient-specific" computational fluid dynamics (CFD) simulations. *IEEE Trans. Med. Imaging* **32**, 153-164 (2012).

2 Piccinelli, M., Veneziani, A., Steinman, D. A., Remuzzi, A. & Antiga, L. A framework for geometric analysis of vascular structures: application to cerebral aneurysms. *IEEE Trans. Med. Imaging* **28**, 1141-1155 (2009).

3 Dhar, S. *et al.* Morphology parameters for intracranial aneurysm rupture risk assessment. *Neurosurgery* **63**, 185 (2008).

4 Katritsis, D. *et al.* Wall shear stress: theoretical considerations and methods of measurement. *Prog. Cardiovasc. Dis.* **49**, 307-329 (2007).

5 He, X. & Ku, D. N. Pulsatile flow in the human left coronary artery bifurcation: average conditions. (1996).

6 Sunderland, K., Huang, Q., Strother, C. & Jiang, J. Two Closely Spaced Aneurysms of the Supraclinoid Internal Carotid Artery: How Does One Influence the Other? *J. Biomech. Eng.* **141** (2019).

7 Sunderland, K. *et al.* Quantitative analysis of flow vortices: differentiation of unruptured and ruptured medium-sized middle cerebral artery aneurysms. *Acta Neurochir. (Wien.)* **163**, 2339-2349 (2021).

8 Haralick, R. M., Shanmugam, K. & Dinstein, I. H. Textural features for image classification. *IEEE Transactions on systems, man, and cybernetics*, 610-621 (1973).

9 Galloway, M. M. Texture analysis using gray level run lengths. *Computer graphics and image processing* **4**, 172-179 (1975).

10 Thibault, G., Angulo, J. & Meyer, F. Advanced statistical matrices for texture characterization: application to cell classification. *IEEE Trans. Biomed. Eng.* **61**, 630-637 (2013).

11 Parekh, V. & Jacobs, M. A. Radiomics: a new application from established techniques. *Expert review of precision medicine and drug development* **1**, 207-226 (2016).

12 Yasaka, K. *et al.* Precision of quantitative computed tomography texture analysis using image filtering: a phantom study for scanner variability. *Medicine* **96** (2017).

13 Daubechies, I. *Ten lectures on wavelets*. (SIAM, 1992).

14 Daubechies, I. Orthonormal bases of compactly supported wavelets. *Communications on pure and applied mathematics* **41**, 909-996 (1988).

15 Haar, A. *Zur theorie der orthogonalen funktionensysteme*. (Georg-August-Universitat, Gottingen., 1909).

16 Mallat, S. G. A theory for multiresolution signal decomposition: the wavelet representation. *IEEE transactions on pattern analysis and machine intelligence* **11**, 674-693 (1989).

17 Van Griethuysen, J. J. *et al.* Computational radiomics system to decode the radiographic phenotype. *Cancer Res.* **77**, e104-e107 (2017).

18 Chaddad, A., Daniel, P. & Niazi, T. Radiomics evaluation of histological heterogeneity using multiscale textures derived from 3D wavelet transformation of multispectral images. *Front. Oncol.* **8**, 96 (2018).

19 Zhou, J. *et al.* Predicting the response to neoadjuvant chemotherapy for breast cancer: wavelet transforming radiomics in MRI. *BMC Cancer* **20**, 1-10 (2020).
